# Multiplexed chemogenetics in astrocytes and motoneurons restore blood–spinal cord barrier in ALS

**DOI:** 10.26508/lsa.201900571

**Published:** 2020-09-08

**Authors:** Najwa Ouali Alami, Linyun Tang, Diana Wiesner, Barbara Commisso, David Bayer, Jochen Weishaupt, Luc Dupuis, Phillip Wong, Bernd Baumann, Thomas Wirth, Tobias M Boeckers, Deniz Yilmazer-Hanke, Albert Ludolph, Francesco Roselli

**Affiliations:** 1 Department of Neurology, Ulm University, Ulm, Germany; 2 International Graduate School in Molecular Medicine Ulm, Ulm, Germany; 3 Department of Neurology, Clinical Neuroanatomy, Ulm University, Ulm, Germany; 4 German Center for Neurodegenerative Diseases (DZNE), Ulm, Germany; 5 CEMMA Graduate School, Ulm University, Ulm, Germany; 6 Inserm U1118, Mécanismes Centraux et Périphériques de la Neurodégénérescence; Université de Strasbourg, Faculté de Médecine, Strasbourg, France; 7 Department of Pathology, Johns Hopkins University School of Medicine, Baltimore, MD, USA; 8 Department of Neuroscience, Johns Hopkins University School of Medicine, Baltimore, MD, USA; 9 Institute of Physiological Chemistry, Ulm University, Ulm, Germany; 10 Department of Anatomy and Cell Biology, Ulm University, Ulm, Germany

## Abstract

Blood–spinal cord barrier (BSCB) disruption is thought to contribute to motoneuron (MN) loss in amyotrophic lateral sclerosis (ALS). It is currently unclear whether impairment of the BSCB is the cause or consequence of MN dysfunction and whether its restoration may be directly beneficial. We revealed that *SOD1*^*G93A*^, *FUS*^Δ*NLS*^, *TDP43*^*G298S*^, and *Tbk1*^*+/−*^ ALS mouse models commonly shared alterations in the BSCB, unrelated to motoneuron loss. We exploit PSAM/PSEM chemogenetics in *SOD1*^*G93A*^ mice to demonstrate that the BSCB is rescued by increased MN firing, whereas inactivation worsens it. Moreover, we use DREADD chemogenetics, alone or in multiplexed form, to show that activation of Gi signaling in astrocytes restores BSCB integrity, independently of MN firing, with no effect on MN disease markers and dissociating them from BSCB disruption. We show that astrocytic levels of the BSCB stabilizers Wnt7a and Wnt5a are decreased in *SOD1*^*G93A*^ mice and strongly enhanced by Gi signaling, although further decreased by MN inactivation. Thus, we demonstrate that BSCB impairment follows MN dysfunction in ALS pathogenesis but can be reversed by Gi-induced expression of astrocytic Wnt5a/7a.

## Abstract (precis)

Chemogenetic motoneuron excitation and astrocyte GPCR-Gi signaling restore blood–spinal cord barrier, disrupted in four ALS mouse models, revealing its role in disease progression but not initiation.

## Introduction

Disruption of the blood–spinal cord barrier (BSCB) is a recently appreciated feature of amyotrophic lateral sclerosis (ALS), a disease affecting upper and lower motoneurons (MNs) with a progressive course and invariably fatal outcome (Hardiman et al, 2017). In spinal cord samples from ALS patients, BSCB impairment has been detected in the form of plasma protein leakage and reduced expression of endothelial tight junction (TJ) proteins (Garbuzova-Davis et al, 2012). Likewise, extravasation of erythrocytes, immunoglobulins and plasma proteins; loss of endothelial TJ proteins (zonula occludens-1 [ZO-1], occludin, and claudin-5 [Zhong et al, 2008]); and decreased astrocytic end-feet and pericyte coverage (Garbuzova-Davis et al, 2007a, 2007b) have been detected in *SOD1*^*G37R*^, *SOD1*^*G85R*^, and *SOD1*^*G93A*^ (Zhong et al, 2008; Winkler et al, 2014) as well as in *SOD1*^*G93A*^ rats (Garbuzova-Davis et al, 2007a, 2007b) even at presymptomatic stages.

Nevertheless, the driver(s) of BSCB disruption in ALS and its ultimate impact on disease progression are still debated. It has been hypothesized that damage of the BSCB may arise as a cell autonomous consequence of mutant SOD1 accumulation in endothelial cells: endothelial cells isolated from *SOD1*^*G93A*^ mice and immortalized mouse endothelial cells expressing the human *G93A* mutant *SOD1* display reduced levels of claudin-5 (CLN-5) and decreased transendothelial resistance (Meister et al, 2015).

Finally, the impact of the BSCB disruption on disease onset and progression is not univocal. It has been suggested that the extravasation of blood-derived factors, inflammatory cells, erythrocytes, and hemoglobin degradation products in itself is able to trigger the degeneration of MN. Indeed, administration of activated protein C (APC [Zhong et al, 2009]) or of iron chelators (Winkler et al, 2014) to *SOD1*^*G93A*^ mice reduced the BSCB leakage and delayed disease progression. On the other hand, warfarin (an anticoagulant interfering with multiple vitamin K–dependent coagulation factors) aggravated BSCB disruption and worsened the degeneration of MN (Winkler et al, 2014). Nevertheless, none of these interventions are selective and may affect multiple disease cascades at once (e.g., reducing the transcription of the mutant *SOD1* gene in the case of APC; Zhong et al, 2009).

Here, we have addressed the origin of the BSCB dysfunction in ALS and its weight on disease initiation and progression exploiting multiplexed chemogenetic strategies (involving both PSAM/PSEM^308^ and DREADDs; Magnus et al, 2011; Roth, 2016) to enable interventions with high cell specificity and precise temporal control. We have demonstrated that BSCB disruption is a consequence of early, excitation-related MN dysfunction and that BSCB integrity can be restored (through the induction of Wnt proteins) by driving Gi signaling in astrocytes. Furthermore, we have used multiplexed chemogenetics to achieve a complete dissociation of BSCB integrity and disease burden, demonstrating how multiplexed chemogenetic can be used to untangle in vivo complex cellular interactions in BSCB disruption.

## Results

### Structural and functional disruption of the blood–spinal cord barrier is common to SOD1, FUS, TDP-43, and Tbk1 ALS mouse models

We set out to investigate the extent and progression of BSCB disruption in four murine ALS models characterized by distinct genetic mutations and different degrees of phenotype severity and progression rate. In particular, we considered the high-copy *SOD1*^*G93A*^ line, together with the *FUS*^Δ*NLS/+*^ (Scekic-Zahirovic et al, 2017), the *TDP-43*^*G298S*^ (Wiesner et al, 2018), and *Tbk1* heterozygous knockout (*Tbk1*^+/−^ [Brenner et al, 2019]) lines. In each ALS mouse strain, we investigated three time points at which critical pathological milestones were reached: (i) high-copy *SOD1*^*G93A*^ mice were studied at P20 (appearance of ER stress and misfolded SOD1 buildup; Saxena et al, 2013), P50 (denervation of highly vulnerable fast fatigable MN; Pun et al, 2006), and P80 (appearance of overt neurological signs; Boillée et al, 2006; Ouali Alami et al, 2018); (ii) *FUS*^Δ*NLS/+*^ mice were studied at P150 (before appearance of neurological signs and denervation), P270 (appearance of denervation and clasping), and P450 (more advanced neurological signs; Scekic-Zahirovic et al, 2017); (iii) *TDP-43*^*G298S*^ mice were studied at P150 (appearance of substantial neurological abnormalities; Wiesner et al, 2018), P360 (plateau of neurological dysfunction), and P510 (later stage in neurological dysfunction; subtle neurological abnormalities are present from the earliest time point but start worsening at around P150 with a plateau at around P250; Wiesner et al, 2018); and (iv) *Tbk1*^*+/−*^ mice were used as a reference line for ALS disease at P270 and P450, when they showed lack of motor symptoms, weight loss, or premature death (Brenner et al, 2019).

**Figure S1.**
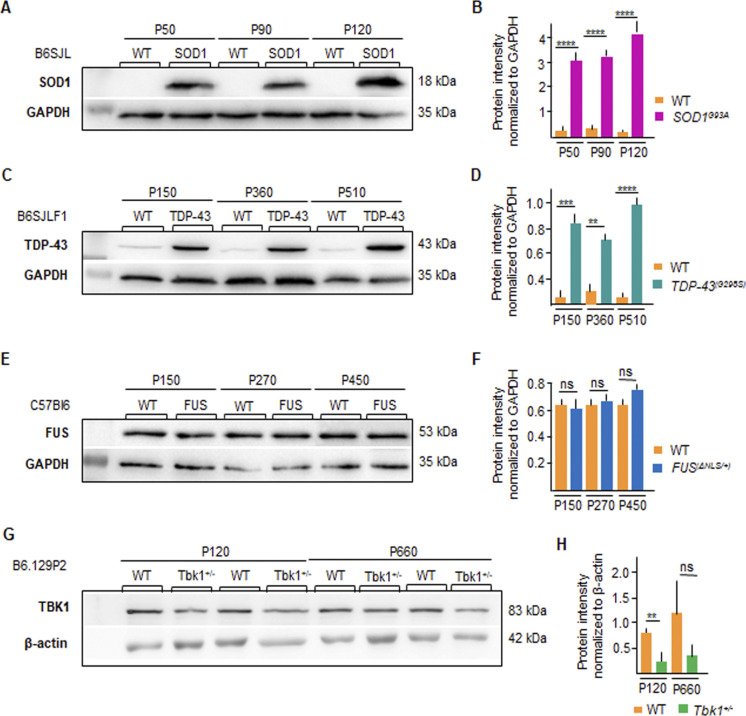
Stability of mutant protein expression in amyotrophic lateral sclerosis mouse models over time. **(A, B)** The expression of the mutant human SOD1 in *SOD1*^*G93A*^ transgenic mice (B6SJL background) is stable across the P50, P90, and P120 time points (normalized for GAPDH). **(C, D)** Persistent and comparable protein expression of the mutant *TDP-43* transgene in *TDP-43*^*G298S*^ (B6SJLF1 background) at P150, P360, and P510 (normalized for GAPDH). **(E, F)** Stable protein expression of mutant FUS in the spinal cord lysate of knock-in *FUS*^Δ*NLS/+*^ and WT mice; as expected, the expression of the mutant FUS is comparable with that of WT-FUS in the WT littermates. **(G, H)** Levels of TBK1 are persistently reduced in *Tbk1*^*+/−*^ mice (normalized for actin). Data information: in (B, D, F, H), data are presented as means ± SD. **P < 0.01, ***P < 0.001, ****P < 0.0001 (two-way ANOVA [genotype × time point] with Bonferroni correction for multiple comparisons).

First, we verified that the expression of the human mutant *SOD1* transgene and the expression of the mutant *TDP-43* transgene were persistently overexpressed and comparable across all time points (Fig S1A–D). Likewise, we verified that in knock-in *FUS*^Δ*NLS/+*^, the FUS protein was not overexpressed (as expected, Scekic-Zahirovic et al, 2016) but stayed stable over time (Fig S1E and F). Finally, TBK1 levels were steadily reduced in *Tbk1*^*+/−*^ mice compared with WT mice (Fig S1G and H).

**Figure S2.**
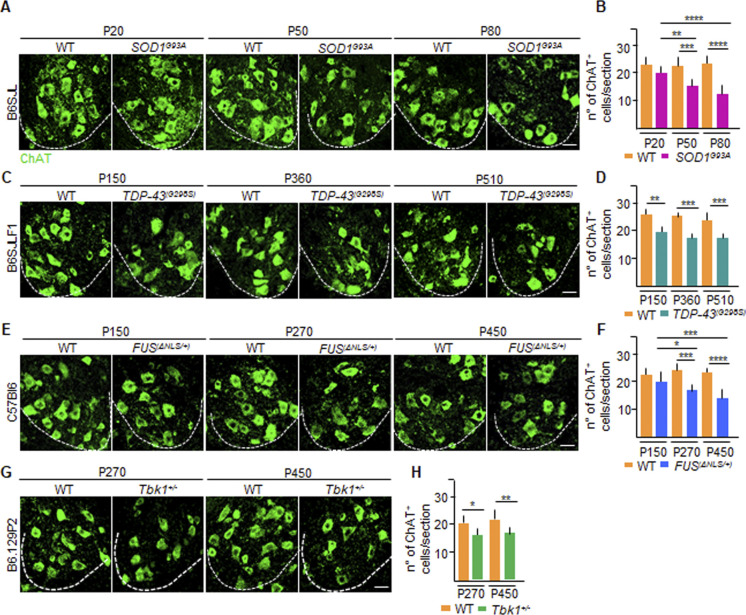
Quantification of motoneuron loss in *SOD1*, *FUS*, *TDP-43*and *Tbk1* amyotrophic lateral sclerosis mouse models. **(A, C, E, G)** Representative pictures of MN counts over time in the ventral horn of the lumbar spinal cord of (A) P20, P50, and P80 WT and *SOD1*^*G93A*^ mice (B6SJL background); (C) P150, P360, and P510 WT and *TDP-43*^*G298S*^ mice (B6SJLF1 background); (E) P150, P270, and P450 WT and *FUS*^Δ*NLS/+*^mice (C57BI6 background); and (G) P270 and P450 WT and *Tbk1*^*+/−*^ mice (B6.129P2 background). MNs are identified by choline acetyltransferase (ChAT) immunostaining (green). White dotted lines mark the border between white and gray matter. **(B, D, F, H)** Quantification of the ChAT^+^ cell number per section in the ventral horn of the lumbar spinal cord from (B) WT and transgenic *SOD1*^*G93A*^ mice (N = 4) at P20, P50, and P80; (D) WT and *TDP-43*^*G298S*^ mice (N = 3) at P150, P360, and P510; (F) WT and *FUS*^Δ*NLS/+*^ mice (N = 3) at P150, P270, and P450; and WT and *Tbk1*^*+/−*^ mice (N = 3) at P270 and P450 (n = 6 sections per mouse). Scale bars: 10 μm. Data information: in (B, D, F, H), data are presented as means ± SD. *P < 0.01, **P < 0.001, ***P < 0.0001, ****P < 0.0001 (two-way ANOVA [genotype × time point] with Bonferroni correction for multiple comparisons).

Next, we characterized the extent of MN loss over time in the four ALS mouse lines by quantifying the number of choline acetyltransferase (ChAT)–positive cells in the ventral horn of the spinal cord. The *SOD1*^*G93A*^ MN contingent was comparable with WT at P20 (average number of MN per lumbar spinal cord section: 21.2 ± 0.8 versus 22.4 ± 3.2, respectively) but steeply declined at P50 (15.7 ± 1.6 versus 22.8 ± 2.5 MN per section, respectively, *P* < 0.001), and was further aggravated at P80 (12.8 ± 2.3 versus 23.2 ± 2.7 MN per section, respectively; *P* < 0.0001; *SOD1*^*G93A*^ P20 versus *SOD1*^*G93A*^ P50: *P* < 0.01; *SOD1*^*G93A*^ P20 versus *SOD1*^*G93A*^ P80: *P* < 0.0001; Fig S2A and B; as previously reported: Ouali Alami et al, 2018). In the *TDP-43*^*G298S*^ animals, a small loss of MN was detected already at P150 (19.6 ± 2.1 versus 26.0 ± 1.7 MN per section; *TDP-43*^*G298S*^ versus WT; *P* < 0.01; Fig S2C and D) but did not significantly worsen at later time points (at P360: 17.4 ± 1.1 versus 25.3 ± 1.7 MN per section; *P* < 0.001 and at P510: 17.2 ± 1.6 versus 24.0 ± 3.1 MN per section; *P* < 0.001 *TDP-43*^*G298S*^ versus WT, respectively; Fig S2C and D). In *FUS*^Δ*NLS/+*^ mice, the MN number was remarkably similar in mutant and WT animals at P150 (20.2 ± 2.2 versus 21.3 ± 1.5 MN per section, Fig S2E and F), but a significant MN loss appeared at P270 (16.3 ± 1.5 versus 22.0 ± 1.1 MN per section, respectively; *P* < 0.001; Fig S2E and F; as previously reported: Scekic-Zahirovic et al, 2017) which did not worsen further at P450 (14.0 ± 3.1 MN per section in *FUS*^Δ*NLS/+*^ versus 21.4 ± 1.1 MN per section in WT; *P* < 0.0001, *FUS*^Δ*NLS/+*^ P150 versus *FUS*^Δ*NLS/+*^ P270: *P* < 0.1; *FUS*^Δ*NLS/+*^ P150 versus *FUS*^Δ*NLS/+*^ P450: *P* < 0.001; Fig S2E and F). In the case of *Tbk1*^*+/−*^ mice, the MN number was decreased at P270 compared with WT (16.8 ± 1.9 versus 20.3 ± 1.9 MN per section; *P* < 0.1; Fig S2G and H) but did not display a further decline at P450 (17.3 ± 1.6 versus 21.7 ± 2.1 MN per section, respectively; *P* < 0.01; Fig S2G and H). Thus, whereas the *SOD1*^*G93A*^ displays a precipitous loss of MN, the *TDP-43*^*G298S*^ and *Tbk1*^*+/−*^ mice display a stable loss of a small contingent of MNs, whereas the *FUS*^Δ*NLS/+*^ mice show a late onset with limited MN loss.

**Figure 1.**
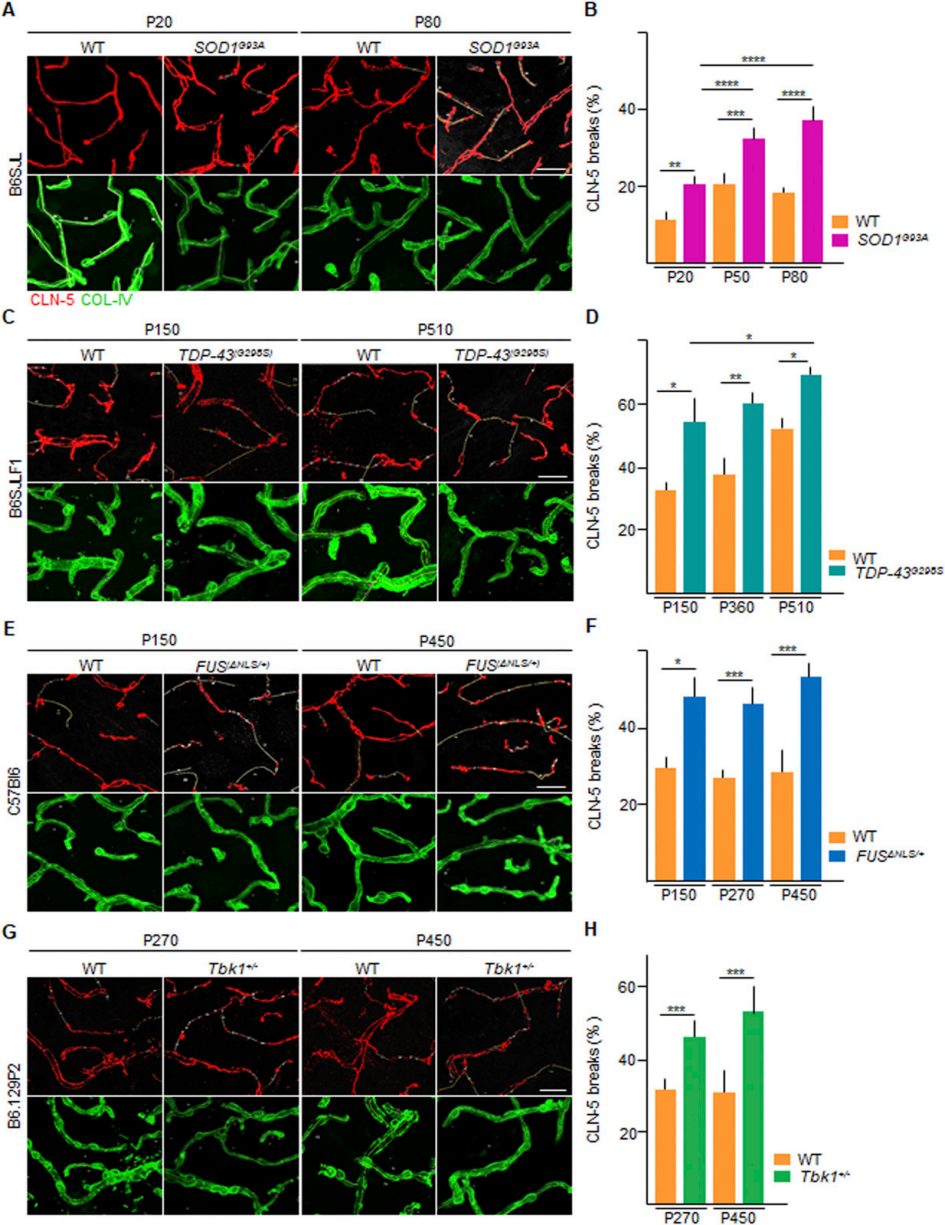
Altered claudin-5 distribution in the spinal cord microvessels of *SOD1*, *FUS*, *TDP-43* and *Tbk1* amyotrophic lateral sclerosis mouse models. **(A, C, E, G)** Representative pictures of ventral horn of the lumbar spinal cord sections stained for claudin-5 (CLN-5 [red]) and collagen-IV (COL-IV [green]) of (A) P20 and P80 WT and *SOD1*^*G93A*^ mice (B6SJL background), (C) P150 and P510 WT and *TDP-43*^*G298S*^ mice (B6SJLF1 background), (E) P150 and P450 WT and *FUS*^Δ*NLS/+*^mice (C57BI6 background), and (G) P270 and P450 WT and *Tbk1*^*+/−*^ mice (B6.129P2 background). CLN-5 breaks are indicated by yellow lines (drawn with ImageJ software), underlining the discontinuity of CLN-5 ribbon. **(B, D, F, H)** Quantification of the capillary bed length (identified by COL-IV immunostaining) devoid of CLN-5 ribbon-like immunolabeling (break length) in the ventral horn of the spinal cord capillaries from (B) WT and transgenic *SOD1*^*G93A*^ mice (N = 4) at P20, P50, and P80; (D) WT and *TDP-43*^*G298S*^ mice (N = 3) at P150, P360, and P510; (F) WT and *FUS*^Δ*NLS/+*^ mice (N = 3) at P150, P270, and P450; and WT and *Tbk1*^*+/−*^ mice (N = 3) at P270 and P450. The quantifications are expressed as % of the total vessel length (n = 6 sections per mouse). Scale bars: 20 μm. Data information: in (B, D, F, H), data are presented as means ± SD. *P < 0.01, **P < 0.001, ***P < 0.0001, ****P < 0.0001 (two-way ANOVA [genotype × time point] with Bonferroni correction for multiple comparisons).

**Figure 2.**
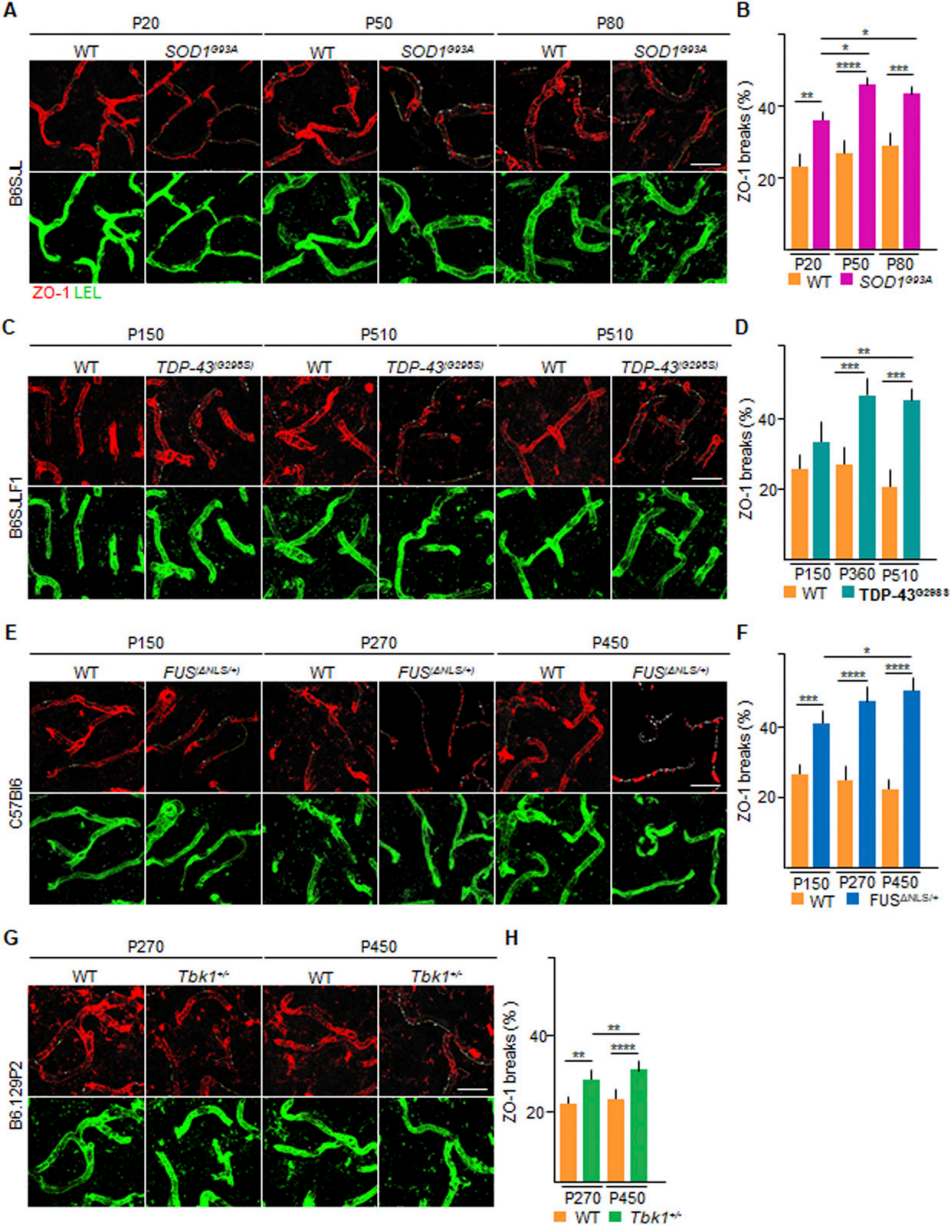
Altered zonula occludens-1 (ZO-1) distribution in the spinal microvessels of *SOD1*, *FUS*, *TDP-43* and *Tbk1* amyotrophic lateral sclerosis mouse models. **(A, C, E, G)** Representative pictures of ventral horn of the lumbar spinal cord sections stained for ZO-1 (red) and *Lycopersicon esculentum* (tomato) lectin (LEL [green]) of (A) P20, P50, and P80 WT and *SOD1*^*G93A*^ mice (B6SJL background); (C) P150, P360, and P510 WT and *TDP-43*^*G298S*^ mice (B6SJLF1 background); (E) P150, P270, and P450 WT and *FUS*^Δ*NLS/+*^mice (C57BI6 background); and (G) P270 and P450 WT and *Tbk1*^*+/−*^ mice (B6.129P2 background). ZO-1 breaks are indicated by yellow lines (drawn with ImageJ software), underlining the discontinuity of ZO-1 ribbon. **(B, D, F, H)** Quantification of the capillary bed length (identified by LEL immunostaining) devoid of ZO-1 ribbon-like immunolabeling (break length) in the ventral horn of the spinal cord capillaries from (B) WT and transgenic *SOD1*^*G93A*^ mice (N = 4) at P20, P50, and P80; (D) WT and *TDP-43*^*G298S*^ mice (N = 3) at P150, P360, and P510; (F) WT and *FUS*^Δ*NLS/+*^ mice (N = 3) at P150, P270, and P450; and WT and *Tbk1*^*+/−*^ mice (N = 3) at P270 and P450. The quantifications are expressed as % of the total vessel length (n = 6 sections per mouse). Scale bars: 20 μm. Data information: in (B, D, F, H), data are presented as means ± SD. *P < 0.01, **P < 0.001, ***P < 0.0001, ****P < 0.0001 (two-way ANOVA [genotype × time point] with Bonferroni correction for multiple comparisons).

We then proceeded to assess the extent of structural disruption of the BSCB in the four mouse lines, using as a readout to the extent of microvasculature, displaying focal loss of the TJ protein claudin-5 (CLN-5; Fig 1A–H) and the TJ protein ZO-1 (Fig 2A–H). In WT animals of each different mouse line, CLN-5 immunoreactivity was concentrated in the TJs between endothelial cells. CLN-5 protein forms a continuous ribbon inside the vessel walls (identified by the basal lamina component collagen-IV [COL-IV]; Fig 2A). Upon disruption of the BSCB, observed in concomitance with neuroinflammation or neurodegenerative processes (Ouali Alami et al, 2018), the ribbon of CLN-5 appears fragmented, forming “breaks” in the continuity of the CLN-5 ribbon. Consistent with previous reports (Ouali Alami et al, 2018), *SOD1*^*G93A*^ mice displayed a significantly larger percentage of vessel length displaying CLN-5 breaks than WT littermates (20% ± 2% versus 12% ± 2%; *P* < 0.01) already at P20 (before any MN loss; Fig 1A and B). The extent of CLN-5 disruption of the BSCB further increased at P50 (33% ± 3% versus 21% ± 3%; *SOD1*^*G93A*^ versus WT, respectively; *P* < 0.001) and at P80 (38% ± 4% versus 19% ± 1%; *SOD1*^*G93A*^ versus WT, respectively, *P* < 0.0001; *SOD1*^*G93A*^ P20 versus *SOD1*^*G93A*^ P50: *P* < 0.0001; *SOD1*^*G93A*^ P20 versus *SOD1*^*G93A*^ P80: *P* < 0.0001; Fig 1A and B). In *TDP-43*^*G298S*^ mice, CLN-5 distribution in the BSCB was already compromised at P150 (54.4% ± 8.1% versus 33.4% ± 3.2%; *TDP-43*^*(G298S)*^ versus WT; *P* < 0.1; Fig 1C and D) and had progressively worsened at P360 (60.2% ± 5.4% versus 37.1% ± 8.1%, respectively; *P* < 0.01; Fig 1C and D) and at P510 (70.5% ± 1.9% versus 53.4% ± 3.4%, in *TDP-43*^*(G298S)*^ versus WT, respectively; *P* < 0.1; *TDP-43*^*(G298S)*^ P150 versus *TDP-43*^*(G298S)*^ P510: *P* < 0.1; Fig 1C and D), despite no further loss of MN. In *FUS*^Δ*NLS/+*^ mice, breakdown of the CLN-5 ribbon was detected already at P150 (before signs of MN loss or of neurological impairment; 48.4% ± 7.5% versus 30.5% ± 4.1% in *FUS*^Δ*NLS/+*^ versus WT mice; *P* < 0.1; Fig 1E and F) and became more intense at P270 (47.6% ± 6% versus 24.5% ± 2.1%; *P* < 0.001) and at P450 (52.1% ± 4.4% versus 29.2% ± 9.1% in *FUS*^Δ*NLS/+*^ versus WT littermates; *P* < 0.001; Fig 1E and F). Most notably, we detected a significant disruption of CLN-5 ribbon also in *Tbk1*^*+/−*^ mice at P270 (46.1% ± 6.2% versus 32.6 ± 5.1; in *Tbk1*^*+/−*^ versus WT; *P* < 0.001; Fig 1G and H) with a further increase finally observed at P450 (53.2% ± 9.3% versus 29.0% ± 9.1% in *Tbk1*^*+/−*^ and WT, respectively; *P* < 0.001; Fig 1G and H), although these mice did not display overt signs of disease (Brenner et al, 2019).

To demonstrate that the appearance of CLN-5 ribbon breaks corresponds to a comprehensive disruption of the BSCB organization, we explored the distribution and localization of ZO-1 (Fig 2A–H), an intracellular TJ protein largely unrelated to claudins (Vogelmann & Nelson, 2005). In WT vessels, ZO-1 immunolocalization was similar to CLN-5, displaying a continuous ribbon inside the COL-IV boundary. In the *SOD1*^*G93A*^ mice, at P20, the percentage of vessel length displaying breaks of ZO-1 ribbons was higher than that in the WT littermates (36.3% ± 2.1% versus 23.8% ± 4.5%, respectively; *P* < 0.01; Fig 2A and B). At P50, the capillary disruption is reflected with a further increase in the percentage of ZO-1 breaks (46.0% ± 1.7% of breaks in the *SOD1*^*G93A*^ compared with 26.5% ± 4.6% in the WT littermate; *P* < 0.0001; Fig 2A and B). This increment has also been observed at P80 (44.1% ± 2.9% versus 29.2% ± 2.7% in *SOD1*^*G93A*^ versus WT, respectively, *P* < 0.001; *SOD1*^*G93A*^ P20 versus *SOD1*^*G93A*^ P50: *P* < 0.1; *SOD1*^*G93A*^ P20 versus *SOD1*^*G93A*^ P80: *P* < 0.1; Fig 2A and B). In the *TDP-43*^*G298S*^ animals, ZO-1 fragmentation was slightly higher at P150 than WT littermates (32.4% ± 4.9% versus 25.5% ± 4.1%, respectively; Fig 2C and D), and significantly increased at P360 (47.6% ± 4.5% versus 27.5% ± 5.1%, respectively; *P* < 0.001; Fig 2C and D) and at P510 (45.1% ± 3.4% versus 20.1% ± 5.1%, respectively; *P* < 0.001; *TDP-43*^*(G298S)*^ P150 versus *TDP-43*^*(G298S)*^ P360: *P* < 0.01; Fig 2C and D). In *FUS*^Δ*NLS/+*^ mice, ZO-1 distribution was already significantly compromised at P150 (40.3% ± 4.1% versus 25.8% ± 2.1% *FUS*^Δ*NLS/+*^ versus WT, *P* < 0.001; Fig 2E and F) and further increased at P270 (46.8% ± 5.7% versus 25.6% ± 4.1% in *FUS*^Δ*NLS/+*^ versus WT; *P* < 0.0001, Fig 2E and F). The extent of ZO-1 ribbon fragmentation further worsened at P450 in *FUS*^Δ*NLS/+*^ mice (50.3% ± 4.3% versus 23.6% ± 2.4% in *FUS*^Δ*NLS/+*^ versus WT; *P* < 0.0001; *FUS*^Δ*NLS/+*^ P150 versus *FUS*^Δ*NLS/+*^ P450: *P* < 01; Fig 2E and F). Remarkably, we also detected loss of ZO-1 in the *Tbk1*^*+/−*^ ALS mouse model at P270 (27.0% ± 0.6% versus 22.7% ± 0.7%; *Tbk1*^*+/−*^ versus WT; *P* < 0.01; Fig 2G and H), with a significant increase at P450 (31.4% ± 2.2% versus 23.5% ± 1.6%, respectively, in *Tbk1*^*+/−*^ versus WT; *P* < 0.0001; *Tbk1*^*+/−*^ P270 versus *Tbk1*^*+/−*^ P450: *P* < 0.01; Fig 2G and H). Overall, these data suggest that fragmentation of TJ protein complexes is crucial in the maintenance of BSCB integrity and occurred before and independently of MN loss in the ALS mice considered.

**Figure 3.**
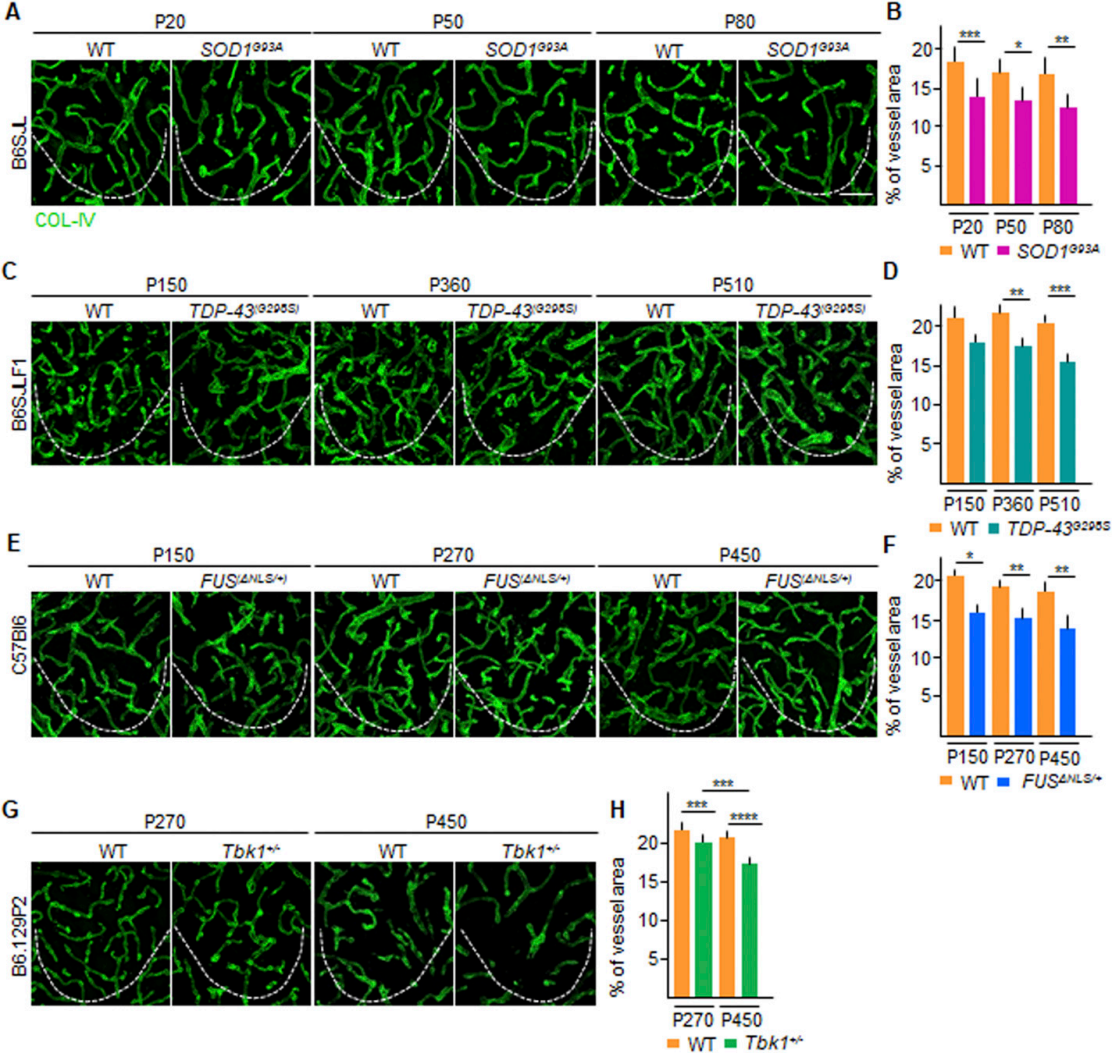
Overall vascular density in *SOD1*, *FUS*, *TDP-43* and *Tbk1* amyotrophic lateral sclerosis mouse models. **(A, C, E, G)** Representative pictures of microvasculature ramification in the ventral horn of the lumbar spinal cord of (A) P20, P50, and P80 WT and *SOD1*^*G93A*^ mice (B6SJL background); (C) P150, P360, and P510 WT and *TDP-43*^*G298S*^ mice (B6SJLF1 background), (E) P150, P270, and P450 WT and *FUS*^Δ*NLS/+*^mice (C57BI6 background); and (G) P270 and P450 WT and *Tbk1*^*+/−*^ mice (B6.129P2 background). The microvessel walls are stained with collagen-IV (COL-IV [green]), specific for type 4 collagen in the basal lamina. **(B, D, F, H)** Quantification of the COL-IV^+^ area in the ventral horn of the spinal cord from (B) WT and transgenic *SOD1*^*G93A*^ mice (N = 4) at P20, P50, and P80; (D) WT and *TDP-43*^*G298S*^ mice (N = 3) at P150, P360, and P510; (F) WT and *FUS*^Δ*NLS/+*^ mice (N = 3) at P150, P270, and P450; and WT and *Tbk1*^*+/−*^ mice (N = 3) at P270 and P450. The quantifications are expressed as % of COL-IV^+^ area per total area (n = 6 sections per mouse). Scale bars: 10 μm. Data information: in (B, D, F, H), data are presented as means ± SD. *P < 0.01, **P < 0.001, ***P < 0.0001, ****P < 0.0001 (two-way ANOVA [genotype × time point] with Bonferroni correction for multiple comparisons).

We wondered whether the loss of TJ proteins may be related to an ongoing degeneration of the spinal cord microvascular bed. We assessed the density of microvessels in the ventral horn of spinal cord samples derived from the *SOD1*^*G93A*^, *FUS*^Δ*NLS/+*^, *TDP43*^*(G298S)*^, and *TBK1*^*+/−*^ mice at different time points and compared them with age- and strain-matched controls. At their baseline, all ALS mice already displayed a degree (∼20% across the different lines) of reduced COL-IV^+^ microvessel density in the ventral horn of the spinal cord. More precisely, at P20 in the *SOD1*^*G93A*^, the percentage of the COL-IV^+^ area in the region of interest (ROI) was decreased (14.2% ± 1.4%) compared with WT littermates (18.4% ± 1.3%; *SOD1*^*G93A*^ versus WT; *P* < 0.001; Fig 3A and B), which persisted unmodified at P50 (14.0% ± 2.0% versus 17.4% ± 1.2% in *SOD1*^*G93A*^ versus WT mice, respectively; *P* < 0.1; Fig 3A and B) with a slight decrease at P80 (12.6% ± 2.4% versus 17.0% ± 1.6%; *P* < 0.01; Fig 3A and B). A similarly stable decrease in vascular density was also detected in *TDP-43*^*G298S*^ mice at P150 (17.6% ± 1.5% versus 20.8% ± 2.2% in *TDP-43*^*G298S*^ versus WT; Fig 3C and D), followed by a significant decrease at P360 (17.3% ± 1.1% versus 21.1% ± 1.4%, respectively; *P* < 0.01; Fig 3C and D) and later at P510 (15.6% ± 1.1% versus 20.7% ± 1.3% in *TDP-43*^*G298S*^ versus WT, respectively; *P* < 0.001; Fig 3C and D). Notably, a significant decrease in vascular density was also detected in *FUS*^Δ*NLS/+*^ mice at P150 (16.6% ± 1.1% versus 20.6% ± 0.6% in *FUS*^Δ*NLS/+*^ versus WT littermates; *P* < 0.1; Fig 3E and F), at P270 (15.6% ± 1.5% versus 19.2% ± 1.6%; *P* < 0.01; Fig 3E and F), and at P450 (14.6% ± 2.3% versus 19.1% ± 1.5%; *P* < 0.01; Fig 3E and F). Despite the absence of neurological abnormalities and MN loss, *Tbk1*^*+/−*^ mice also displayed alteration in the vascular density (Fig 3G and H), with a significant reduction in the COL-IV^+^ area at P270 and at P450 (20.0% ± 1.1% and 17.0% ± 1.8%, respectively; *P* < 0.001), in contrast to WT littermates (23.0% ± 1.1% and 21.1% ± 1.6%, respectively; *Tbk1*^*+/−*^ P270 versus WT P270 *P* < 0.001; *Tbk1*^*+/−*^ P450 versus WT P450 *P* < 0.0001; Fig 3G and H).

**Figure S3.**
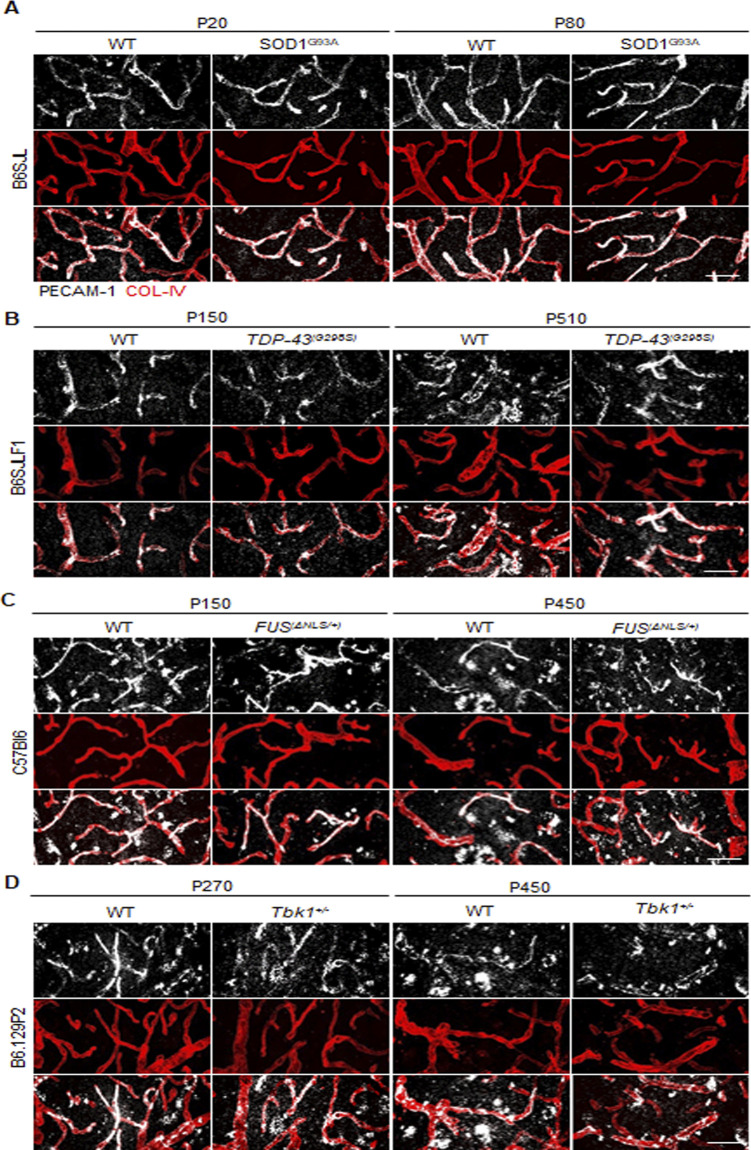
PECAM-1^+^ endothelial cells line all COL-IV+ vessels in the spinal cord of *SOD1*, *FUS*, *TDP-43* and *Tbk1* amyotrophic lateral sclerosis mouse models. **(A, B, C, D)** Representative pictures of microvasculature ramification in the ventral horn of the lumbar spinal cord of (A) P20 and P80 in WT and *SOD1*^*G93A*^ mice (B6SJL background), (B) P150 and P510 in WT and *TDP-43*^*G298S*^ mice (B6SJLF1 background), (C) P150 and P450 in WT and *FUS*^Δ*NLS/+*^mice (C57BI6 background), and (D) P270 and P450 in WT and *Tbk1*^*+/−*^ mice (B6.129P2 background). The microvessel walls are stained with collagen-IV (COL-IV [red]), specific for type 4 collagen in the basal lamina and PECAM-1 (white) specific for defining endothelial origin. Analysis is from N = 3 mice per group of experiments. Scale bars: 20 μm.

We further explored the possibility that TJ loss may be due to the focal degeneration of endothelial cells (in the so-called string vessel formation, defined as a collapsed basement membrane without the endothelium and with no function in circulation; Forsberg et al, 2018). We immunostained spinal cord sections for COL-IV^+^ together with CD31/PECAM-1, a membrane and cytoplasmic protein abundantly and constitutively expressed on endothelial cells (Feng et al, 2004; Caligiuri, 2019). Across all ALS lines and all time points, we observed an almost complete (>90%) coverage of COL-IV^+^ with CD31/PECAM-1^+^ immunostaining, confirming that every COL-IV^+^ vessel was indeed lined with endothelial cells (Fig S3).

**Figure 4.**
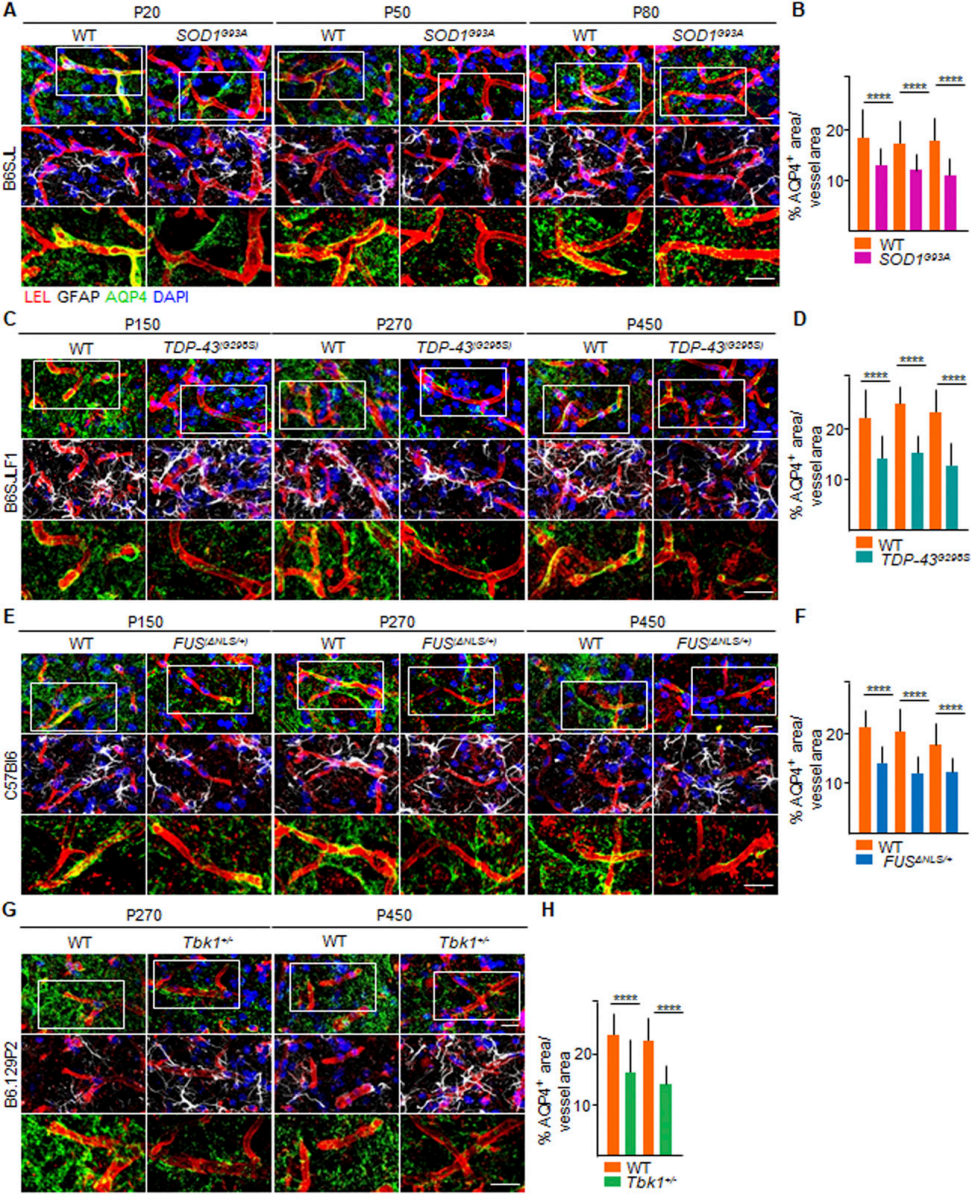
Altered aquaporin-4^+^ astrocytic end-feet coverage of spinal cord vessels in *SOD1*, *FUS*, *TDP-43* and *Tbk1* amyotrophic lateral sclerosis mouse models. **(A, C, E, G)** Representative images of astrocytic end-feet coverage around the vessels (red), immunostained with aquaporin-4 (AQP4 [green]) in the ventral horn of the lumbar spinal cord of (A) P20, P50, and P80 WT and *SOD1*^*G93A*^ mice (B6SJL background); (C) P150, P360, and P510 WT and *TDP-43*^*G298S*^ mice (B6SJLF1 background); (E) P150, P270, and P450 WT and *FUS*^Δ*NLS/+*^mice (C57BI6 background); and (G) P270 and P450 WT and *Tbk1*^*+/−*^ mice (B6.129P2 background). The first row shows the overlap of AQP4 surrounding the vessels, labeled with *Lycopersicon esculentum* (tomato) lectin (LEL [red]); the second row shows the extremity of astrocytes, immunolabeled with glial fibrillary acidic protein (white), wrapping the capillaries (red). Nuclei are detected by DAPI staining (blue); the third row represents a high magnification of the insert in the first row (white rectangle) and displays the distribution of AQP4 on the LEL^+^ endothelium, as a marker of blood–spinal cord barrier stability. The magnified pictures do not include DAPI staining. Scale bar: 20 μm. **(B, D, F, H)** Quantification of the AQP4^+^ area, expressed as % of the total vessel area in the ventral horn of the spinal cord from (B) WT and transgenic *SOD1*^*G93A*^ mice (N = 4) at P20, P50, and P80; (D) WT and *TDP-43*^*G298S*^ mice (N = 3) at P150, P360, and P510; (F) WT and *FUS*^Δ*NLS/+*^ mice (N = 3) at P150, P270, and P450; and WT and *Tbk1*^*+/−*^ mice (N = 3) at P270 and P450; (n = 6 sections per mouse). Scale bars: 10 μm. Data information: in (B, D, F, H), data are presented as means ± SD. ****P < 0.0001 (two-way ANOVA [genotype × time point] with Bonferroni correction for multiple comparisons).

Moreover, we assessed a third readout of BSCB integrity, namely, the extent of astrocytic end-feet vessel coverage (Kubotera et al, 2019). To this end, we immunostained spinal cord sections from the four ALS lines with aquaporin-4 (AQP4), a water channel protein, highly expressed in astrocytic end-feet (Nielsen et al, 1997), and glial fibrillary acidic protein (GFAP), a class-III intermediate filament and cell-specific marker that distinguishes astrocytes from other glial cells, combined with COL-IV. Whereas spinal cord vessels in WT mice (of any strain) displayed substantial coverage with AQP4^+^ processes, ALS mice exhibit a relevant decrease in AQP4^+^ coverage. Indeed *SOD1*^*G93A*^ mice at P20 displayed a % of AQP4^+^ area equal to 13.20% ± 3.8% of the total vessel area, compared with 18.8% ± 4.6% of the total vessel area in corresponding WT littermates (*P* < 0.0001; Fig 4A and B). AQP4 coverage stayed stable either at P50 (12.1% ± 3.9% versus 17.8% ± 4.2% in *SOD1*^*G93A*^ versus WT; *P* < 0.0001, Fig 4A and B) or at P80 (11.3% ± 4.1% versus 18.3% ± 4.5% of the total vessel area, respectively; *P* < 0.0001; Fig 4A and B). In *TDP-43*^*G298S*^ mice, the AQP4^+^ area was already significantly decreased at P150 (14.0% ± 4.3% of the vessel area) compared with WT littermates (22.1% ± 4.2% of the total vessel area; *P* < 0.0001; Fig 4C and D), despite no further decrease over time point P360 (15.0% ± 2.8% versus 24.1% ± 3.7% of the total vessel area, in *TDP-43*^*G298S*^ mice versus WT, respectively; *P* < 0.0001; Fig 4C and D) and P510 (12.6% ± 3.9% versus 22.6% ± 5.0% of the total vessel area in *TDP-43*^*G298S*^ mice versus WT littermates; *P* < 0.0001; Fig 4C and D). In *FUS*^Δ*NLS/+*^ mice at P150, we also witnessed a conspicuous reduction in the astrocytic water channel AQP4 surrounding the blood microvessels (14.3% ± 3.6% of the total vessel area), in contrast to WT mates (21.0% ± 3.7%; *P* < 0.0001; Fig 4E and F). The amount of AQP4 lining *FUS*^Δ*NLS/+*^ vessels did not diminish over time point P270 (12.1% ± 3.5% versus 20.6% ± 4.5%; *FUS*^Δ*NLS/+*^ versus WT, respectively; *P* < 0.0001; Fig 4E and F) and P450 (12.6% ± 2.5% versus 19.7% ± 4.7%; *FUS*^Δ*NLS/+*^ versus WT, respectively; *P* < 0.0001; Fig 4E and F). The same was observed in *Tbk1*^*+/−*^ mice at P270 (*Tbk1*^*+/−*^ versus WT, *P* < 0.0001) and P450 (*Tbk1*^*+/−*^ versus WT, *P* < 0.0001; Fig 4G and H).

**Figure S4.**
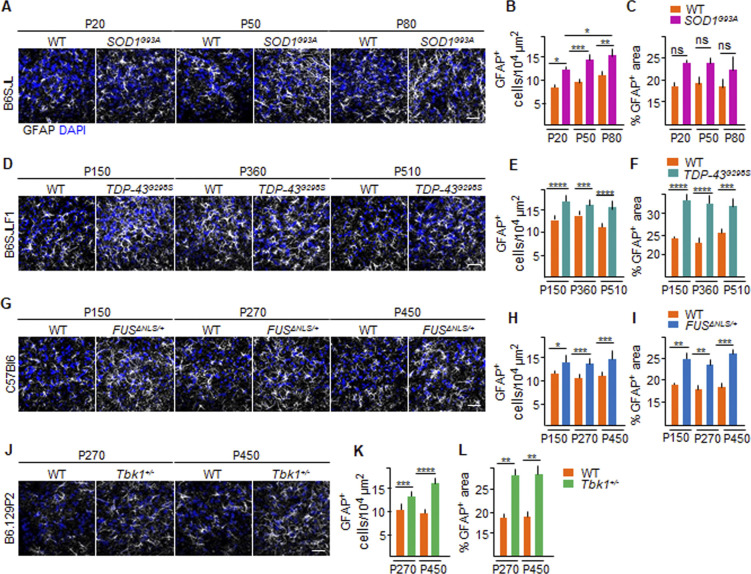
Astrocytes density in the ventral horn of the spinal cord from *SOD1*, *FUS*, *TDP-43* and *Tbk1*. **(A, D, G, J)** Representative pictures of astrocytic distribution, identified by immunolabeling GFAP^+^ cells and area (white) in the ventral horn of the lumbar spinal cord of (A) P20, P50, and P80 WT and *SOD1*^*G93A*^ mice; (D) P150, P360, and P510 WT and *TDP-43*^*G298S*^ mice (B6SJLF1 background); (G) P150, P270, and P450 WT and *FUS*^Δ*NLS/+*^mice (C57BI6 background); and (J) P270 and P450 WT and *Tbk1*^*+/−*^ mice (B6.129P2 background). Nuclei are stained with DAPI (blue). **(B, E, H, K)** Number of GFAP^+^ cells per area (10^4^ μm). **(B, C, D, F, I, L)** Quantification of % GFAP^+^ area versus total ventral horn area in the lumbar spinal cord from (B) WT and transgenic *SOD1*^*G93A*^ mice (N = 4) at P20, P50, and P80; (D) WT and *TDP-43*^*G298S*^ mice (N = 3) at P150, P360, and P510; (F) WT and *FUS*^Δ*NLS/+*^ mice (N = 3) at P150, P270, and P450; and WT and *Tbk1*^*+/−*^ mice (N = 3) at P270 and P450; (n = 6 sections per mouse). The values are expressed as % of the GFAP^+^ area per total area. Scale bars: 10 μm. Data information: in (B, C, E, F, H, I, K, L), data are presented as means ± SD. *P < 0.01, **P < 0.001, ***P < 0.0001, ****P < 0.0001 (two-way ANOVA [genotype × time point] with Bonferroni correction for multiple comparisons).

This effect cannot be attributed to a loss of astrocytes because an actual increase in the cell number (astrocyte hyperplasia; Fig S4B, E, H, and K) and size (hypertrophy, as measured by cumulative GFAP^+^ area; Fig S4C, F, I, and L) was detected in the four ALS lines, either progressively increasing in the *SOD1*^*G93A*^ mouse (i.e., P20: 12.4 ± 0.6 versus 9.6 ± 0.3 GFAP^+^ cell/10^4^ μm in *SOD1*^*G93A*^ versus WT; *P* < 0.1; P50: 14.5 ± 1.1 versus 10.1 ± 0.4 GFAP^+^ cell/10^4^ μm, respectively; *P* < 0.001; P80: 15.3 ± 1.9 versus 11.3 ± 0.4 GFAP^+^ cell/10^4^ μm, respectively; *P* < 0.01; *SOD1*^*G93A*^ P20 versus *SOD1*^*G93A*^ P80: *P* < 0.1; Fig S4A and B), as previously reported (Ouali Alami et al, 2018) or stably increasing over the time points investigated for the *TDP43* (Fig S4D and E), *FUS* (Fig S4G and H), and *Tbk1* (Fig S4J and K) mice. The loss or retraction of AQP4^+^ processes thus appears to be an additional aspect of the BSCB dysfunction.

**Figure 5.**
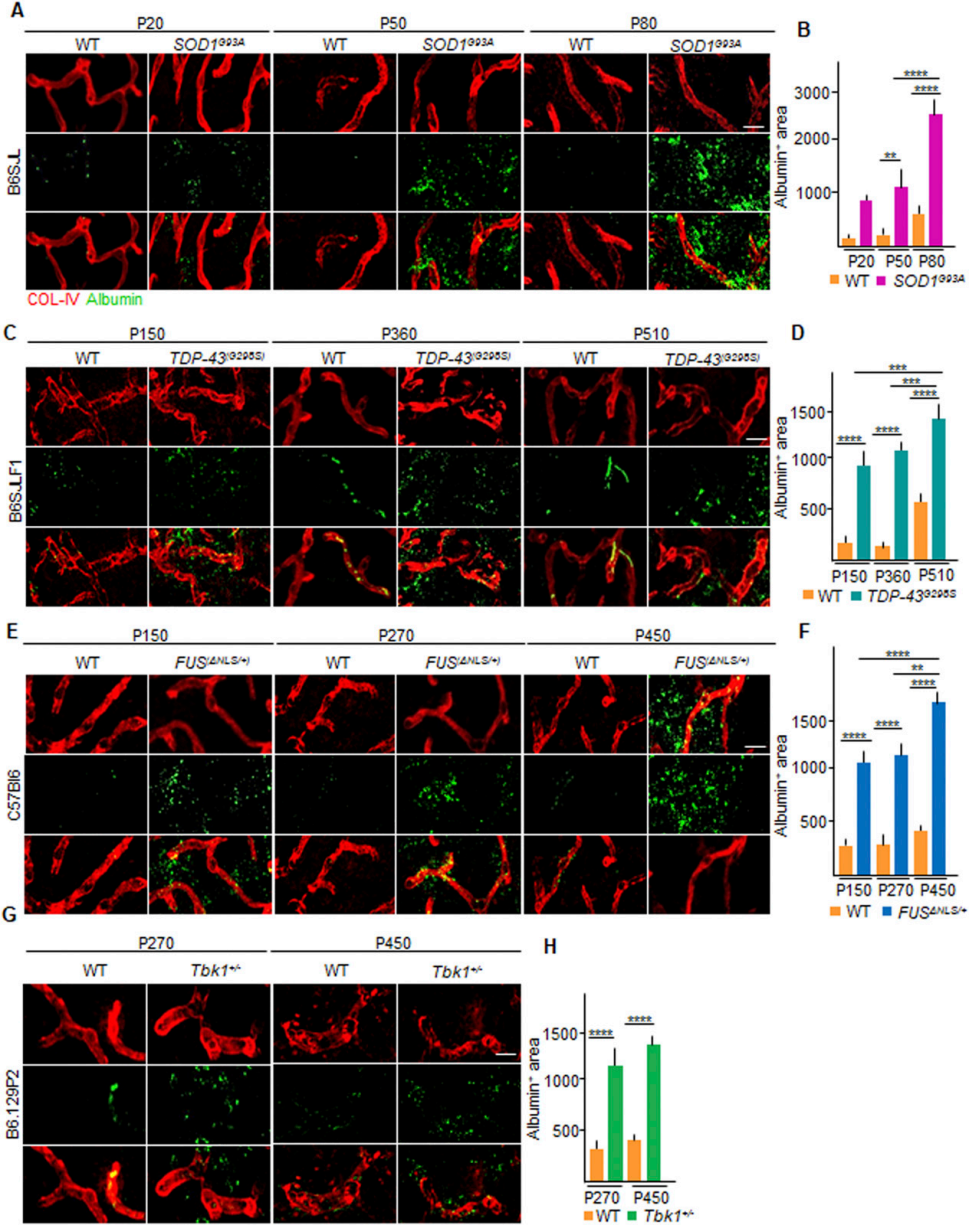
Albumin extravasation demonstrates functional impairment of the blood–spinal cordbarrier in SOD1, FUS, TDP-43, and Tbk1 amyotrophic lateral sclerosis mouse models. **(A, C, E, G)** Representative images displaying intraparenchymal albumin staining (green) around the vessels (COL-IV; red) localized in the ventral horn of the lumbar spinal cord of (A) P20, P50, and P80 WT and *SOD1*^*G93A*^ mice (B6SJL background); (C) P150, P360, and P510 WT and *TDP-43*^*G298S*^ mice (B6SJLF1 background); (E) P150, P270, and P450 WT and *FUS*^Δ*NLS/+*^mice (C57BI6 background); and (G) P270 and P450 WT and *Tbk1*^*+/−*^ mice (B6.129P2 background). The first row shows vessels immunostained with COL-IV (red) alone. The second row shows the distribution of albumin (green) in the parenchyma in the ventral horn of the spinal cord. The third row represents images merged from the two previous images. **(B, D, F, H)** Quantification of the area covered by albumin in the ventral horn of the spinal cord from (B) WT and transgenic *SOD1*^*G93A*^ mice (N = 4) at P20, P50, and P80; (D) WT and *TDP-43*^*G298S*^ mice (N = 3) at P150, P360, and P510; (F) WT and *FUS*^Δ*NLS/+*^ mice (N = 3) at P150, P270, and P450; and WT and *Tbk1*^*+/−*^ mice (N = 3) at P270 and P450 (n = 6 sections per mouse). Scale bars: 20 μm. Data information: in (B, D, F, H), data are presented as means ± SD. **P < 0.001, ***P < 0.0001, ****P < 0.0001 (two-way ANOVA [genotype × time point] with Bonferroni correction for multiple comparisons).

We completed our evaluation of the BSCB integrity in the ALS mouse lines by assessing the functional impairment of the BSCB by quantifying the amount of extravasated intraparenchymal albumin in the different mouse models. WT animals always displayed limited parenchymal albumin immunoreactivity. On the other hand, in the *SOD1*^*G93A*^ mice, albumin extravasation was already detectable at P20 (mean immunostaining intensity over the parenchymal area: 862 ± 54 versus 173 ± 60 a.u. in *SOD1*^*G93A*^ versus WT; Fig 5A and B) and significantly increased at P50 (1,142 ± 492 versus 240 ± 50 a.u. in *SOD1*^*G93A*^ versus WT; *P* < 0.01; Fig 5A and B) and at P80 (2,466 ± 394 versus 547 ± 287 a.u. *SOD1*^*G93A*^ versus WT, respectively, *P* < 0.0001; *SOD1*^*G93A*^ P50 versus *SOD1*^*G93A*^ P80: *P* < 0.0001; Fig 5A and B). Likewise, in the *TDP-43*^*G298S*^ mice, albumin extravasation was detectable at P150 (914 ± 202 versus 199 ± 103 a.u. in *TDP-43*^*G298S*^ versus WT; *P* < 0.000; Fig 5C and D), with further increase detected at P360 (1,090 ± 83 versus 180 ± 43 a.u., respectively, in *TDP-43*^*G298S*^ versus WT; *P* < 0.000; Fig 5C and D), along with augmentation at P510 (1,428 ± 212 a.u. in *TDP-43*^*G298S*^ versus 568 ± 108 a.u. in WT; *P* < 0.0001; *TDP-43*^*G298S*^ P150 versus *TDP-43*^*G298S*^ P510: *P* < 0.001; *TDP-43*^*G298S*^ P360 versus *TDP-43*^*G298S*^ P510: *P* < 0.001; Fig 5C and D). Furthermore, *FUS*^Δ*NLS/+*^ mice display increased levels of albumin extravasation in comparison with their corresponding WT littermates at P150 (1,025 ± 182 versus 347 ± 103 a.u., respectively; *P* < 0.0001, Fig 5E and F), including at P270 (1,234 ± 194 a.u. versus 399 ± 148 a.u., respectively; *P* < 0.0001; Fig 5E and F) with a substantial increase at P450 (1,657 ± 195 a.u. versus 495 ± 75 a.u. in *FUS*^Δ*NLS/+*^ versus WT, respectively; *P* < 0.0001; *FUS*^Δ*NLS/+*^ P150 versus *FUS*^Δ*NLS/+*^ P450: *P* < 0.0001; *FUS*^Δ*NLS/+*^ P270 versus *FUS*^Δ*NLS/+*^ P450: *P* < 0.01; Fig 5E and F). Similarly, *Tbk1*^*+/−*^ mice albumin extravasation was also significantly increased compared with WT littermates at P270 (1,161.0 ± 400 a.u. versus 338.3 ± 86 a.u., respectively; *P* < 0.0001; Fig 5G and H) and later on at P450 (1,384.0 ± 267 versus 422.0 ± 80 a.u., respectively; *P* < 0.0001; Fig 5G and H).

Consequently, these data show not only that structural and functional BSCB disruption is a generalized phenomenon discovered in all four ALS mouse models but also that BSCB breakdown is already detectable at stages in which there is no MN loss (P20 in *SOD1*^*G93A*^, P150 in *FUS*^Δ*NLS/+*^, and P270 and P450 in *Tbk1*^*+/−*^ mice).

### Chemogenetic inactivation of MN firing enhances BSCB disruption, whereas stimulation of MN excitation restores BSCB integrity

**Figure 6.**
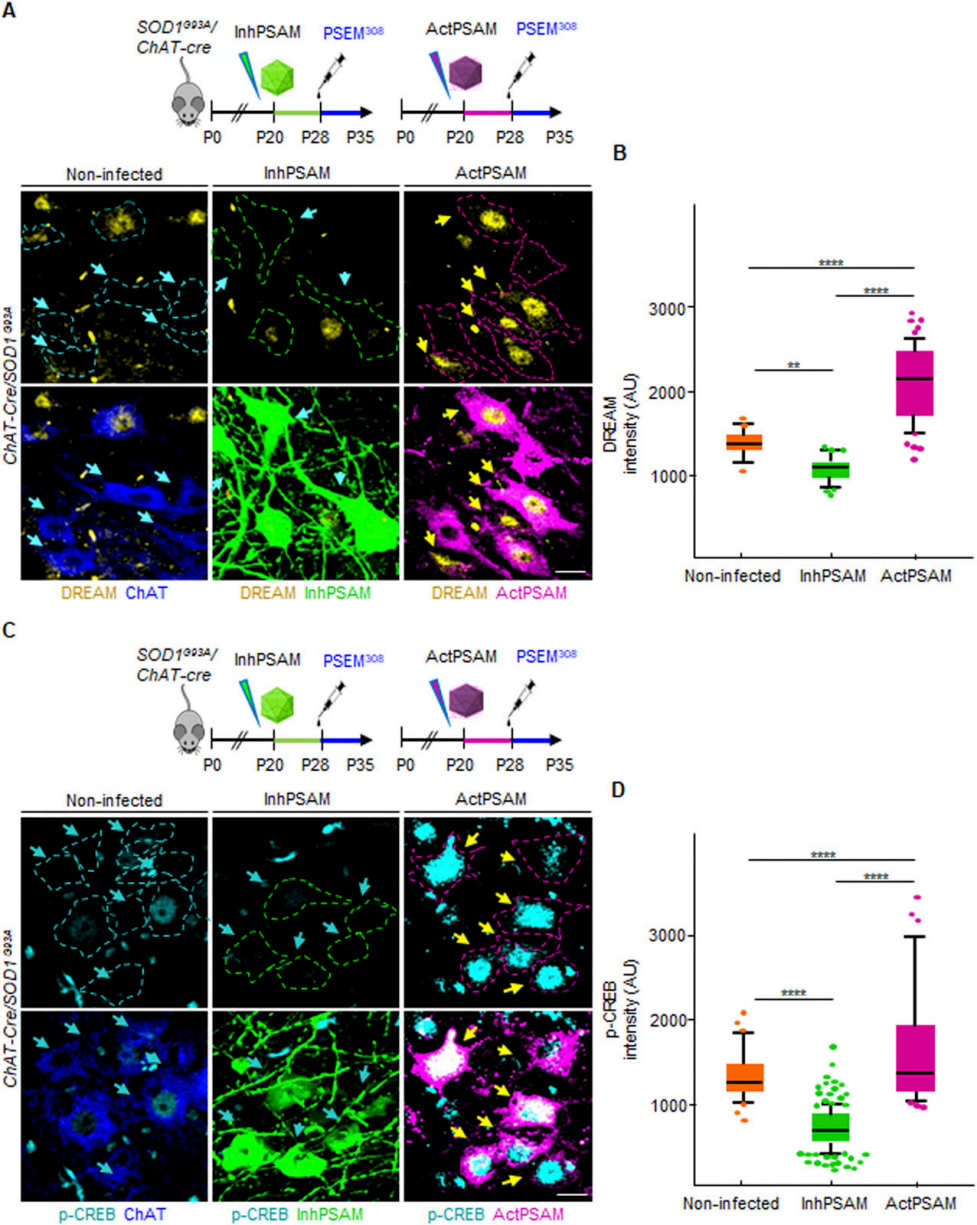
PSAM/PSEM chemogenetics effectively modify MN activity. **(A, C)** Schematic diagram representing the experimental design for control of MN excitation by inhPSAM/PSEM^308^ (green) and actPSAM/PSEM^308^ (magenta) on the assessment on (A) DREAM/KChIP3 (yellow) and (C) p-CREB expression in MNs (cyan blue). Dotted lines delimit the border of MNs, which are further detected by ChAT immunostaining (blue). **(A, C)** Cyan blue arrows indicate the absence or less (A) DREAM/KChIP3 and (C) p-CREB staining in noninfected MN nuclei and in inhPSAM/PSEM^308^-infected MN nuclei (green). **(A, C)** Yellow arrows point to increased levels of (A) DREAM/KChIP3 and (C) p-CREB staining in actPSAM/PSEM^308^-infected MN nuclei. **(B, D)** Quantification of (B) DREAM/KChIP3 and (D) p-CREB intensity, expressed in a.u. in nuclei of noninfected, inhPSAM/PSEM^308^ and actPSAM/PSEM^308^ MNs. The quantifications are represented by box-and-whisker plot; 10–90 percentile is considered. Scale bars: 20 μm. Data are from N = 5 mice per group. Data information: in (B, D), data are presented as means ± SD. **P < 0.001, ****P < 0.0001 (one-way ANOVA with Bonferroni correction for multiple comparisons).

**Figure 7.**
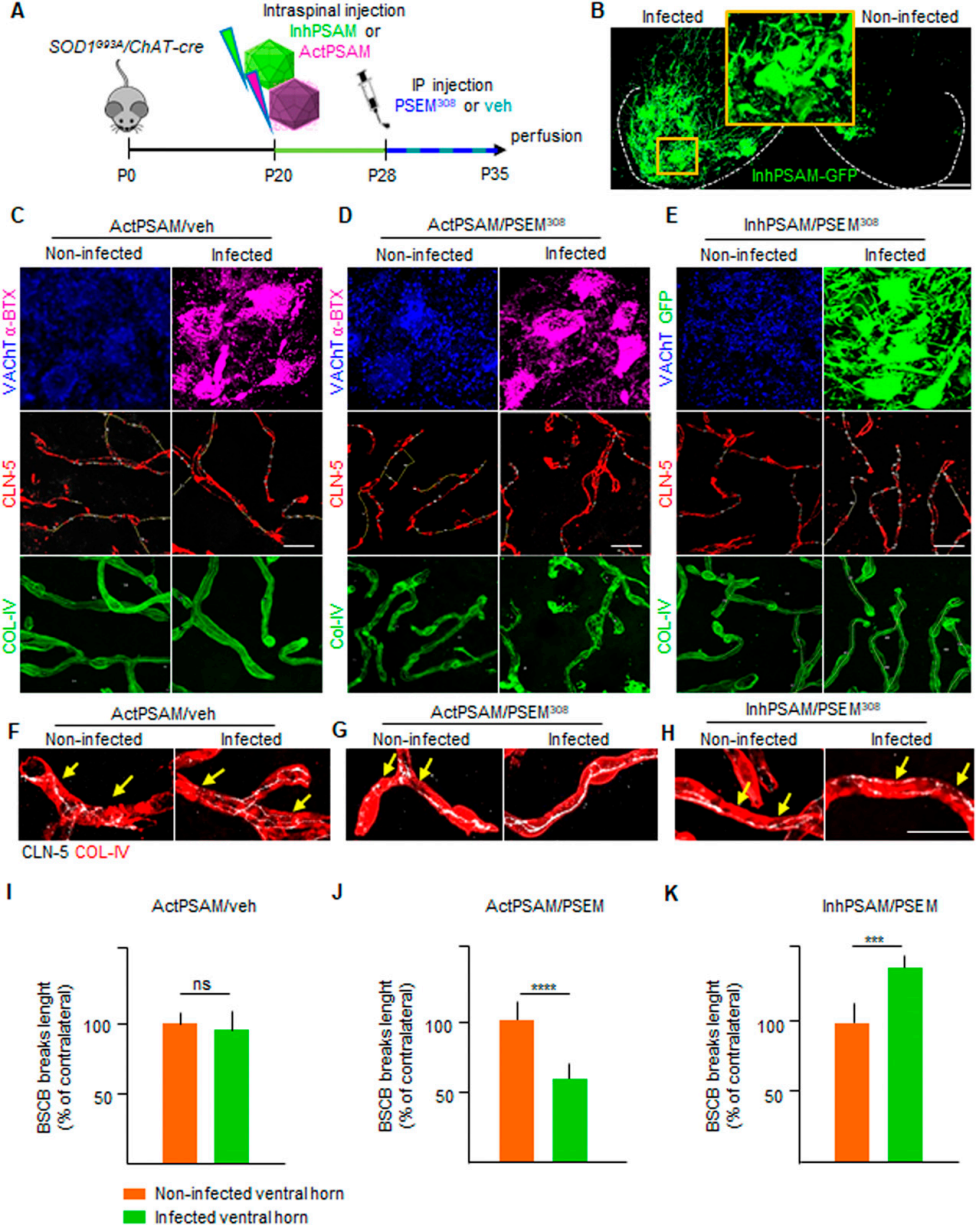
Inhibition of MN firing increases claudin-5 breaks while MN firing enhancement restores blood–spinal cord barrier impairment. **(A)** Experimental design for the chemogenetic control of MN excitation with inhPSAM/PSEM^308^ (green) or actPSAM/PSEM^308^ (magenta) in *SOD1*^*G93A*^*/ChAT-cre* mice injected at P20 and treated with the effector PSEM^308^ from P28 until P35. **(B)** Robust MN expression of the inhPSAM (green) upon intraspinal injection of AAV9 in contrast to no expression in the contralateral uninjected side. Dotted lines delineate the boundaries of gray and white matter in the ventral horns of the spinal cord. Scale bar: 50 μm. **(C)** Panel showing chemogenetic expression of actPSAM/veh (treated with vehicle instead of the effector PSEM^308^) in infected MNs (α-bungarotoxin in magenta) in contrast to uninfected MNs stained with VAChT. The panel shows no difference in cumulative breaks length (yellow lines) in CLN-5 ribbon (red) along microvessels (identified by COL-IV immunostaining in green) in the uninfected ventral horn, in comparison with the contralateral infected ventral horn (N = 4). **(D)** Panel showing chemogenetic expression of actPSAM/PSEM^308^ in infected MNs (α-BTX in magenta) in contrast to uninfected MNs stained with VAChT. Microvessels (green) in proximity of MN activated by actPSAM/PSEM^308^ (magenta) display a reduced cumulative breaks length (yellow) of CLN-5 ribbon (red) compared with contralateral uninfected MNs (N = 6). **(E)** Panel displaying chemogenetic expression of inhPSAM/PSEM^308^ in infected MNs (immunostained with GFP in green) in contrast to uninfected MNs stained with VAChT. Microvessels in proximity of MNs inactivated by inhPSAM/PSEM^308^ (green) display an increase in cumulative breaks length (yellow) of CLN-5 ribbon (red) compared with contralateral uninfected MNs (N = 6). **(F, G, H)** High-magnification view of CLN-5 distribution (white) in the single capillaries (red) of the noninfected and infected ventral horns in inhPSAM/veh, inhPSAM/PSEM^308^, or actPSAM/PSEM^308^. Yellow arrows indicate the breaks in the CLN-5 ribbon. Scale bar: 10 μm. **(I, J, K)** Quantification of blood–spinal cord barrier breaks in uninfected and infected ventral horn of the spinal cord of *SOD1*^*G93A*^*/ChAT-cre* mice subjected to chemogenetic control of activation/inhibition of MN firing. The values are expressed as % of the uninfected contralateral side. Scale bars: 20 μm. Data information: in (I, J, K), data are presented as means ± SD. ***P < 0.0001, ****P < 0.0001 (unpaired *t* test).

We therefore established that BSCB disruption is an early event both shared by ALS mouse lines expressing different pathogenic mutations and appreciable before MN loss (in the *SOD1*^*G93A*^ and *FUS*^Δ*NLS/+*^ mice). We then investigated the pathogenic mechanisms potentially responsible for initiating the BSCB disruption. Loss of excitability in vulnerable MN is among the earliest manifestations of disease (Martinez-Silva et al, 2018), and enhancement of MN excitation has a direct beneficial effect on the burden of misfolded proteins, such as misfolded SOD1, as well as on ER stress and autophagy overload (Saxena et al, 2013; Bączyk et al, 2020). We therefore set out to investigate if early changes in MN excitability may be causally related to the disruption of the BSCB. For this purpose, we exploited an engineered ion channel with orthogonal pharmacology (PSAM/PSEM; [Magnus et al, 2011]), to either stimulate (cation-permeable PSAM: actPSAM) or inactivate (anion-permeable PSAM: inhPSAM) neuronal activity upon administration of the pharmacologically selective synthetic ligand (PSEM^308^ hydrochloride [PSEM^308^]). Intraspinal injections with AAV9 vector (encoding either the actPSAM or the inhPSAM in double-inverted orientation under the human synapsin promoter: hSyn::DIO-actPSAM or hSyn::DIO-inhPSAM.EGFP) were performed in P20 *SOD1*^*G93A*^*/ChAT-Cre* double-tg mice (Fig 7A). Starting from P28, when a robust MN expression of the PSAM was observed, the mice were daily treated for 7 d with PSEM^308^ or with vehicle (Fig 7B). We verified the effectiveness of the chemogenetic modulation on MN firing by showing that actPSAM activation resulted in a significant up-regulation of the activity-dependent gene product DREAM/KChIP3 in the nuclei of infected MNs, compared with the uninfected contralateral MNs (average fluorescence intensity per nucleus: 2,073 ± 475 a.u. versus 1,402 ± 104, respectively; *P* < 0.0001; Fig 6A and B). Likewise, S133-phosphorylated CREB ([pCREB] Wu et al, 2001) was increased in the actPSAM-infected MNs compared with the contralateral uninfected MNs (1,694 ± 723 a.u. versus 1,287 ± 330 a.u., respectively; *P* < 0.0001; Fig 6C and D). Conversely, inhPSAM caused a significant decrease in both activity markers: DREAM (1,043 ± 103 a.u.; inhPSAM versus uninfected; *P* < 0.01; inhPSAM versus actPSAM; *P* < 0.0001, Fig 6A and B) and p-CREB (760 ± 278 a.u.; inhPSAM versus uninfected; *P* < 0.0001; inhPSAM versus actPSAM; *P* < 0.0001; Fig 6C and D) in the infected MNs.

After establishing that chemogenetic approaches have the expected significant impact on the overall activity of infected MNs, we explored the influence of chemogenetic control of MN firing (Fig 7A–K) on the CLN-5 ribbon integrity.

Injection of the AAV9 vector, encoding for the actPSAM, followed by administration of vehicle (instead of the ligand PSEM^308^; Fig 7A and C) did not cause a substantial disruption of the CLN-5 ribbon, compared with the contralateral ventral horn (97.1% ± 20.5% of the uninjected contralateral ventral horn; Fig 7C, F, and I). The experiment proved that intraspinal injection per se did not produce any permanent disruption of the BSCB. Intriguingly, chemogenetic activation of MN firing, by actPSAM/PSEM^308^, caused a significant decrease in the BSCB disruption compared with the uninfected ventral horn, with breaks in CLN-5 ribbon reduced up to 59.3% ± 13.1% of contralateral (*P* < 0.0001; Fig 7D, G, and J). By contrast, the inactivation of MN firing by inhPSAM/PSEM^308^ resulted in increased fragmentation of the CLN-5 ribbon in the infected ventral horn (129.8% ± 12.7% of contralateral), compared with the noninfected contralateral ventral horn (*P* < 0.001; Fig 7E, H, and K; actPSAM/PSEM^308^ versus inhPSAM/PSEM^308^: *P* < 0.0001; Fig 7J and K). These data suggest that BSCB disruption is dependent upon the early excitation-associated dysfunction of MN.

### Chemogenetic activation of astrocytic Gi and Gq signaling is sufficient to restore BSCB integrity

**Figure 8.**
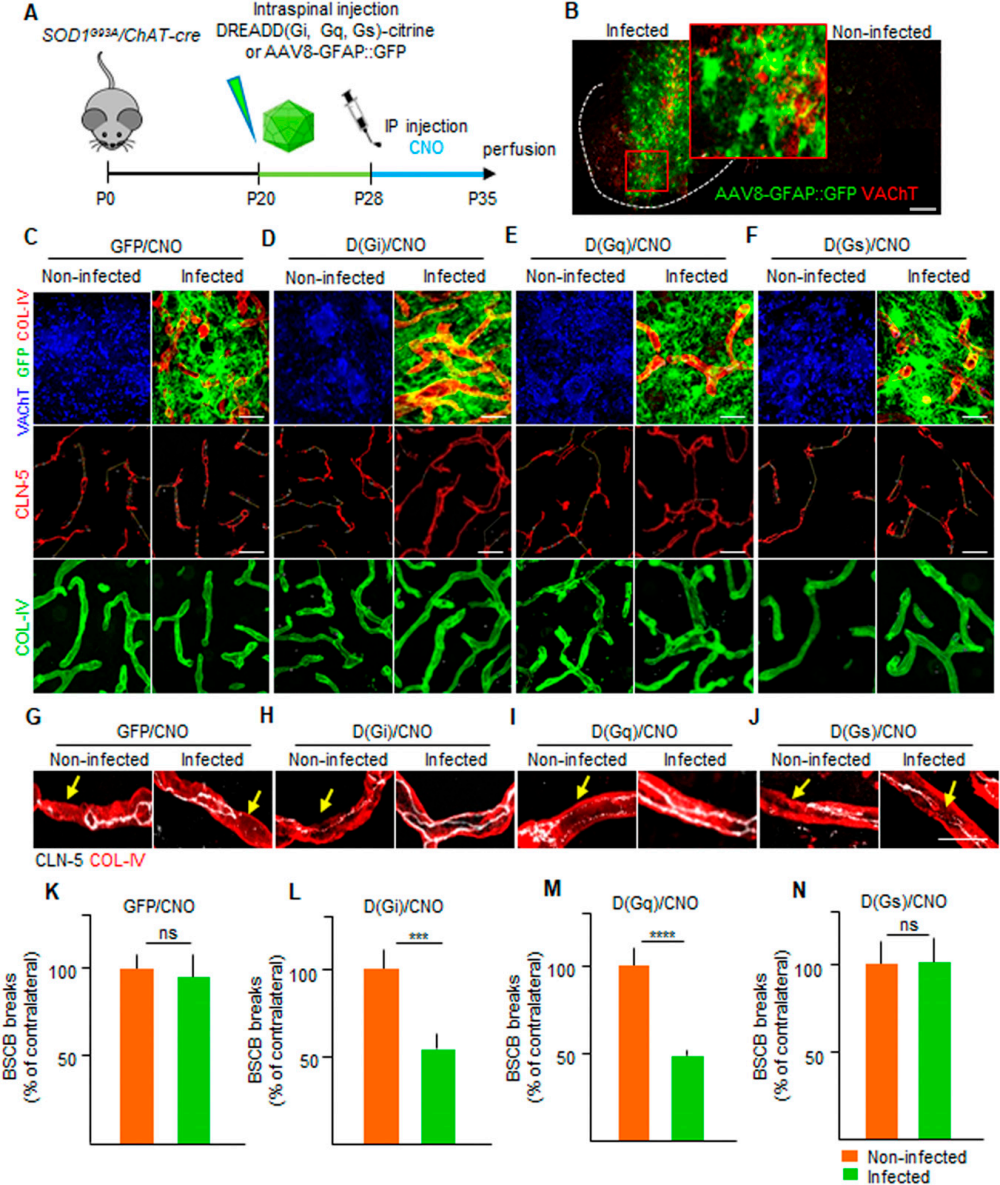
Chemogenetic control of astrocytic Gi and Gq signaling restores blood–spinal cord barrier disruption. **(A)** Experimental design for the injection of AAV8 encoding GFAP::DREADD-GFP, D(Gi), D(Gq), and D(Gs) in *SOD1*^*G93A*^*/ChAT-cre* mice, injected at P20 and treated with the agonist CNO from P28 until P35. **(B)** Pattern of expression of AAV8(GFAP::DREADD-GFP) injected in the ventral horn of the spinal cord and identified by GFP immunostaining (green). The dotted line depicts the boundary of gray and white matter. The insert shows a high magnification of infected astrocytes in the injected ventral horn in contrast to no GFP (astrocytes) staining in the contralateral uninjected ventral horn. Scale bar: 50 μm. **(C)** Panel showing the chemogenetic expression of AAV8(GFAP::GFP) (infected astrocytes in green), followed by CNO administration. The activation does not affect the cumulative breaks length (yellow lines) in CLN-5 ribbon (red) along the COL-IV^+^ vessels (green) in the infected ventral horn of the spinal cord compared with the contralateral uninfected ventral horn. **(D)** Panel displaying the activation of D(Gi)/CNO in astrocytes. The activation of astrocytic Gi signaling causes the reduction of CLN-5 break (yellow lines) burden in the infected horn compared with the contralateral uninfected horn. **(E)** Panel displaying representative pictures of D(Gq)/CNO activation in astrocytes, resulting in decreased CLN-5 break (yellow lines) burden in the infected ventral horn. **(F)** Representative pictures showing activation of D(Gs)/CNO in infected astrocytes. D(Gs) activation does not modify CLN-5 break (yellow lines) burden when compared with the contralateral uninfected ventral horn. **(C, D, E, F)** MNs in the uninjected ventral horn are detected by VAChT immunostaining (blue). Infected astrocytes, identified by GFP (green), are co-stained with COL-IV (red) to visualize the vasculature surrounding the astrocytes in the injected ventral horn. **(G, H, I, J)** High-magnification view of CLN-5 distribution (white) in the single microvessels (red), displaying the effect of control GFP/CNO, D(Gi)/CNO, D(Gq)/CNO, and D(Gs)/CNO activation in infected and noninfected ventral horns. Yellow arrows indicate the breaks in the CLN-5 ribbon. Scale bar: 10 μm. **(K, L, M, N)** Quantification of blood–spinal cord barrier breaks in uninfected and infected ventral horns of the spinal cord of *SOD1*^*G93A*^*/ChAT-cre* mice. Data are from N = 8 mice per group of experiments subjected to injection of AAV8(GFAP::GFP)/CNO, D(Gi)/CNO, D(Gq)/CNO, and D(Gs)/CNO. The values are expressed as % of the uninfected contralateral side. Scale bars: 20 μm. Data information: in (K, L, M, N), data are presented as means ± SD. ***P < 0.0001, ****P < 0.0001 (unpaired *t* test).

The application of chemogenetic PSAM/PSEM^308^ to control MN firing in an ALS mouse model (*SOD1*^*G93A*^) established a causal link between MN activity and stability of the BSCB in ALS. Two sets of considerations led us to hypothesize that astrocytes may provide a link between neuronal firing and the BSCB in an ALS disease condition: (i) astrocytes sense and respond to increased synaptic activity and neuronal firing by detecting the release of (among others) ATP, glutamate, and acetylcholine (Durkee & Araque, 2019; Kofuji & Araque, 2020) as well as K^+^ ions (Simard & Nedergaard, 2004); (ii) astrocytes are involved in the control of BSCB differentiation and integrity (Abbott et al, 2006; Liebner et al, 2018) by releasing GDNF (Igarashi et al, 1999), angiopoietin-1, and bFGF (Lee et al, 2003). Considering that neuronal activity is monitored by astrocytes via different heteromeric G protein–coupled receptor (GPCR) families and signaling through Gα proteins (Gs, Gi, or Gq; Kofuji & Araque, 2020), we hypothesize that controlling the Gs, Gi, or Gq signaling cascade through designer receptors (DREADDS, coupled to Gs [D(Gs)], Gi [D(Gi)], or Gq [D(Gq)]), we could provoke astrocyte responses related to increased neuronal activity and possibly restore the integrity of the BSCB without intervention on MN themselves. We therefore injected AAV8 encoding GFP alone or citrine-tagged DREADDs coupled to Gs, Gi, or Gq under the control of the GFAP mini promoter (Fig 8A and B) in the spinal cord of P20 *SOD1*^*G93A*^*/ChAT-Cre* mice (although the expression of Cre is not necessary to target astrocytes, we used the same line as the PSAM/PSEM^308^ experiments to maintain a comparable background). Clozapine-N-Oxide (CNO, or vehicle) was administered from P28 until P35 (Fig 8A). The integrity of the CLN-5 ribbon was then evaluated by comparing the injected ventral horn of the spinal cord to the contralateral uninjected ventral horn as an internal control (subject to the same treatments and pharmacology but not receiving the virus). Under these conditions, we verified that DREADDs or GFP was expressed in 65–90% of GFAP^+^ cells in the ventral horn of the spinal cord (insert in Fig 8B). When compared with the contralateral ventral horn, the GFP alone in astrocytes (under CNO treatment) did not affect the extent of BSCB breakdown (CLN-5 breaks were 95.8% ± 21.8% of the uninfected contralateral ventral horn; Fig 8C, G, and K). Likewise, expression of DREADDs followed by vehicle treatment did not result in significant changes in BSCB breakdown in injected versus non-injected sides. Taken together, these data confirm that neither CNO treatment nor AAV injection per se affects BSCB integrity. However, when we expressed D(Gi) in astrocytes and administered CNO, the injected side displayed a significant restoration of CLN-5 distribution compared with the contralateral, non-injected side (52.5% ± 12.7% of contralateral; *P* < 0.001; Fig 8D, H, and L). A similar effect was also detected when D(Gq) was activated (49.2% ± 1.3% of the contralateral side; *P* < 0.0001; Fig 8E, I, and M) but not in the case of D(Gs) (103.1% ± 27.8% of the contralateral uninfected side; Fig 8F, J, and N).

**Figure 9.**
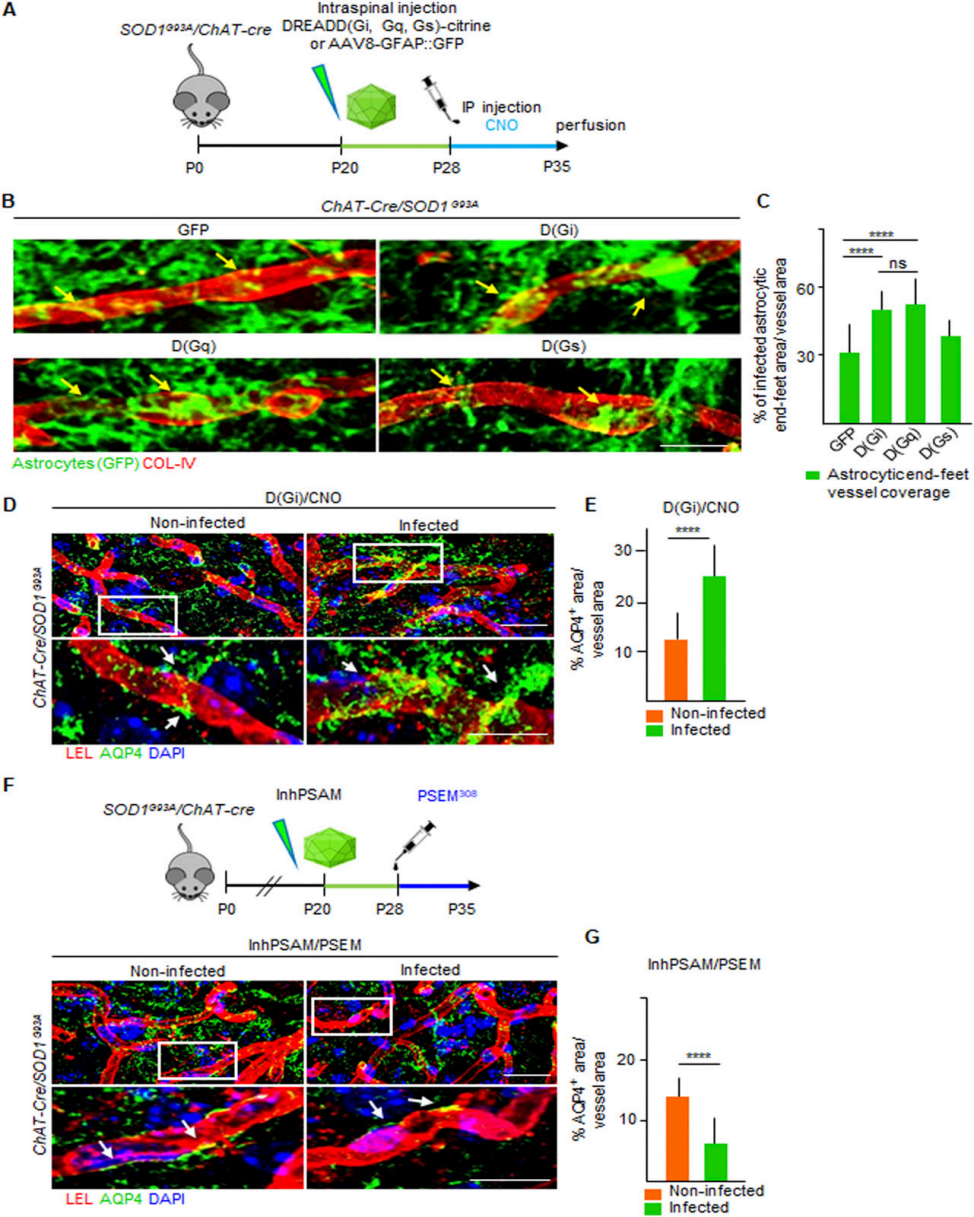
Astrocytic end-feet coverage of microvessels is enhanced by D(Gi) and D(Gq) activation, while diminished after MN firing inhibition. **(A)** Experimental design for the injection of AAV8 encoding GFAP::DREADD-GFP, D(Gi), D(Gq), and D(Gs) in *SOD1*^*G93A*^*/ChAT-cre* mice, injected at P20 and treated with the agonist CNO from P28 until P35. **(B)** High magnification of single microvessels (red) showing astrocytic end-feet coverage (green) after GFP, D(Gi), D(Gq), and D(Gs) activation. The pattern of infected astrocytes (identified by GFP immunostaining in green) on the vessels significantly increases in D(Gi)/CNO, D(Gq)/CNO, and (DGs)/CNO compared with GFP/CNO alone. Scale bar: 7 μm. **(C)** Quantification of end-feet coverage of vessels after DREADD treatments, expressed as % of the GFP^+^ area on the total vessel area. **(D)**. Representative pictures of aquaporin-4 (green) expression localized in the astrocytic end-feet enveloping microvessels (red) in the ventral horn of the spinal cord of SOD1^G93A^/ChAT-cre. Nuclei are immunostained with DAPI (blue). Inserts show high magnification of AQP4 (green) labeling, colocalized with a single microvessel identified by COL-IV (red) staining. Scale bars: 7 μm. **(E)** Quantification of the AQP4^+^ area surrounding the vessels in the *SOD1*^*G93A*^*/ChAT-cre* mice injected with D(Gi)/CNO (N = 3). Values are expressed as % of the total vessel area. **(F)** Experimental design displaying MN firing inactivation via inhPSAM/PSEM^308^ in SOD1^G93A^/ChAT-cre mice. The inhibition of MN firing reduces AQP4 expression at the level of astrocytic end-feet coverage surrounding the vessels, localized in the infected ventral horn compared with the uninfected contralateral horn. Inserts show high magnification of AQP4 immunostaining (green) colocalized with single microvessels (red). White arrows indicate the distribution of AQP4. Scale bar: 7 μm. **(G)** Quantification of the AQP4^+^ area surrounding the vessels in the *SOD1*^*G93A*^*/ChAT-cre* mice injected with inhPSAM/PSEM^308^. Data are from N = 3 mice per group of experiments. Values are expressed as % of the total vessel area. Scale bars: 20 μm. Data information: in (C, E, G), data are presented as means ± SD. **(C, E, G)** ****P < 0.0001 (one-way ANOVA with Bonferroni correction for multiple comparisons [C] and unpaired *t* test [E, G]).

We further evaluated the effect of DREADD signaling activation on the astrocytic end-feet coverage of spinal cord capillaries (Fig 9A–G), an additional parameter to evaluate BSCB integrity. Compared with GFP-expressing astrocytes (Fig 9A–C), activation of D(Gi) resulted in a significant increase in vascular end-feed coverage (51.2% ± 11.8% in D(Gi) versus 30.7% ± 12.0% in GFP-expressing astrocytes; *P* < 0.01; Fig 9B and C). Likewise, D(Gq) also increased end-feet coverage (52.9 ± 13.0; *P* < 0.01 versus GFP; Fig 9B and C), whereas activation of DREADD-(Gs) did not significantly affect the coverage of microvessels by astrocyte end feed (40.5% ± 8.4%; *P* < 0.1; Fig 9B and C). The effect on astrocyte end feet was additionally confirmed by monitoring the levels of aquaporin 4 (AQP4 [Bataveljic et al, 2014]) surrounding the microvessels, located in the ventral horn of the spinal cord. In animals injected with D(Gi) and treated with CNO, the AQP4^+^ coverage of microvessels was strongly increased compared with the contralateral uninjected ventral horn of the spinal cord (25.7% ± 6.8% versus 12.6% ± 4.8%, respectively, *P* < 0.0001; Fig 9D and E). Moreover, we also verified that AQP4^+^ coverage of vessels also responded to chemogenetic control of MN excitability. Indeed, MN inactivation by inhPSAM, resulted in the significant decrease in AQP4^+^ coverage of vessels compared with the contralateral ventral horn (7.7% ± 3.1% versus 12.1% ± 4.1%, respectively; *P* < 0.001; Fig 9F and G).

Taken together, these data show that activation of Gi- and Gq-coupled signaling cascades in astrocytes successfully restores BSCB integrity in presymptomatic *SOD1*^*G93A*^ mice.

### Activation of astrocytic D(Gq) but not D(Gi) ameliorates MN disease markers

**Figure 10.**
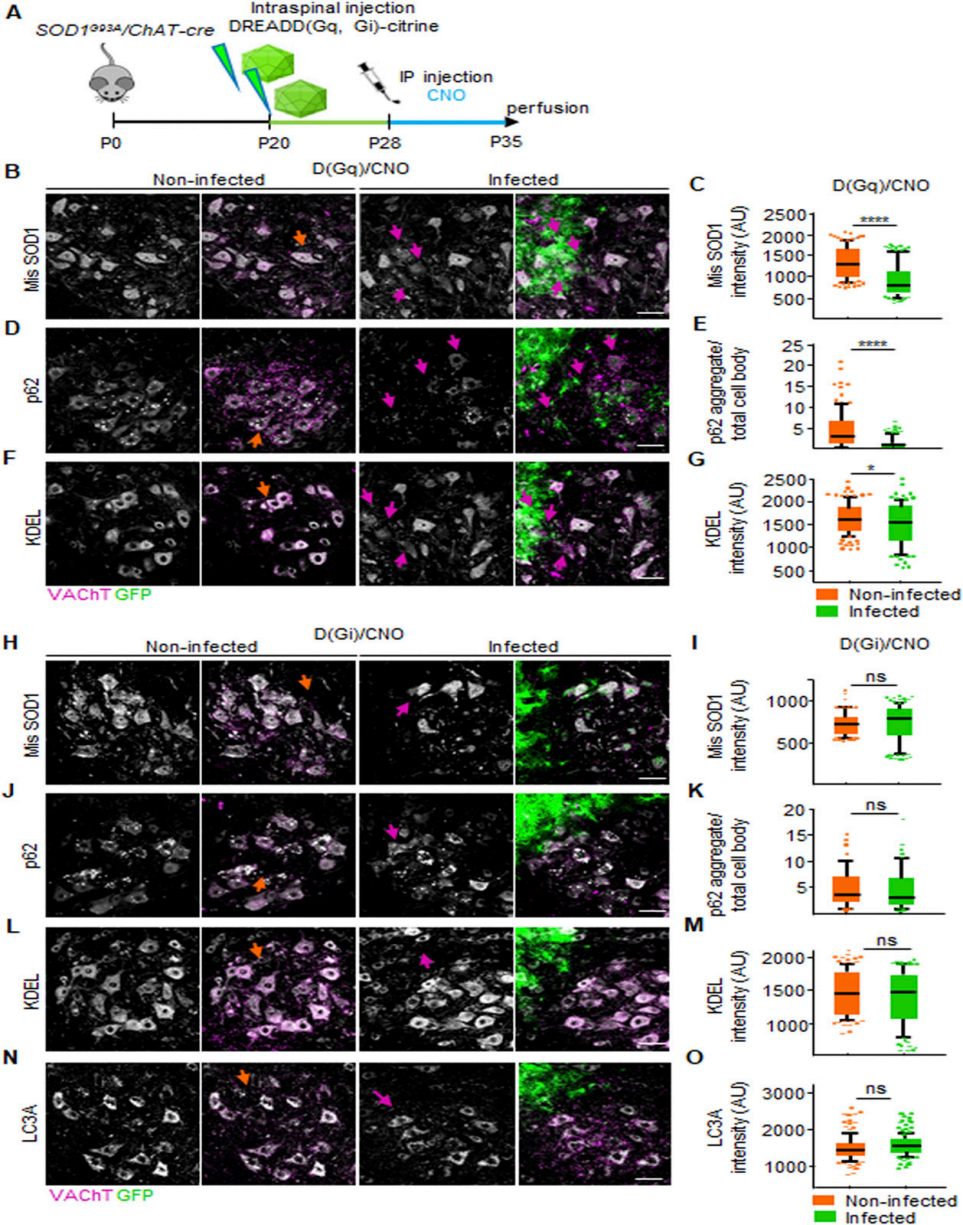
Chemogenetic activation of astrocytic Gq, but not Gi, signaling decreases the burden of disease markers in MN. **(A)** Experimental design for the injection of AAV8 encoding D(Gq) and D(Gi) in *SOD1*^*G93A*^*/ChAT-cre* mice, at P20 and treated with the agonist CNO from P28 until P35. **(B, D, F)** Representative pictures of (B) misfolded SOD1 (white), (D) p62 (white), and (F) KDEL (white) immunostaining in MN located close to the activated astrocytes (green) in the infected ventral horn and in MN in the contralateral uninfected ventral horn of *SOD1*^*G93A*^*/ChAT-cre* spinal cords subjected to AAV8 D(Gq)/CNO injection. MNs are identified by VAChT immunostaining (magenta), and infected astrocytes are detected by GFP (green). **(B, D, F)** Orange arrows indicate the overload of (B) misfolded SOD1 (white), (D) p62 (white), and (F) KDEL (white) burden in the MNs located in the noninfected ventral horn, and magenta arrows point to less accumulation of (B) misfolded SOD1 (white), (D) p62 (white), and (F) KDEL (white) burden in MN close to Gq activated astrocytes (green). **(C, E, G)** Quantification of (C) the intensity of misfolded SOD1, (E) p62 aggregates per total cell body, and (G) KDEL intensity in MNs located in the infected and uninfected contralateral ventral horns of mice subjected to chemogenetic activation of astrocytes via D(Gq)/CNO. **(H, J, L, N)** Representative pictures of (H) misfolded SOD1 (white), (J) p62 (white), (L) KDEL (white), and (N) LC3A (white) immunostaining in MN located close to infected astrocytes (green) in the infected ventral horn and in MNs in the contralateral uninfected ventral horn of *SOD1*^*G93A*^*/ChAT-cre* spinal cords subjected to AAV8 D(Gi)/CNO injection. **(H, J, L, N)** Orange arrows indicate the overload of (H) misfolded SOD1 (white), (J) p62 (white), (L) KDEL (white), and (N) LC3A (white) burden in the MNs located in the noninfected ventral horn, while magenta arrows point to decreased levels of (H) misfolded SOD1 (white), (J) p62 (white), (L) KDEL (white), and (N) LC3A (white) burden in MN close to Gi-activated astrocytes (green). The first column of each experiment shows MN markers alone, and the second column displays the co-immunostaining with VAChT or the co-immunostaining with VAChT and GFP (for infected astrocytes). **(I, K, M, O)** Quantification of (I) the intensity of misfolded SOD1, (K) p62 aggregates per total cell body, (M) KDEL, and (O) LC3A intensity in MNs located in the infected and uninfected contralateral ventral horns of mice subjected to chemogenetic activation of astrocytes via D(Gi)/CNO. The quantifications are represented by a box-and-whisker plot; 10–90 percentile is considered. Data are from N = 6 mice per group of experiments. Scale bars: 20 μm. Data information: in (C, E, G, I, K, M, O), data are presented as means ± SD. *P < 0.1, ****P < 0.0001 (unpaired *t* test).

Next, we tested if the restoration of BSCB integrity by D(Gi) and D(Gq) correlates with beneficial or detrimental effects on the MN burden of established disease markers (Fig 10A). We considered the accumulation of the misfolded protein SOD1 (using the conformation-specific antibody B8H10; Bosco et al, 2010), ER stress levels (KDEL; Saxena et al, 2013), and the burden of p62^+^ inclusions (Rudnick et al, 2017; Martinez-Silva et al, 2018; Ouali Alami et al, 2018). The levels of misfolded SOD1 in MN (magenta arrows in Fig 10B), proximal to D(Gq)-activated astrocytes, were significantly lower than those in contralateral MN (orange arrows in Fig 10B [935 ± 346 versus 1,303 ± 342 a.u., respectively, *P* < 0.0001; Fig 10C]). Likewise, activation of astrocytic D(Gq) decreased the number of p62^+^ inclusions (1.1% ± 1.5% versus 4.3% ± 4.7% aggregate burden/total cell body, *P* < 0.0001; Fig 10D and E) and ER stress (1,495 ± 459 versus 1,621 ± 358 a.u., respectively; *P* < 01; Fig 10F and G) in nearby MNs (magenta arrows), when compared with the contralateral noninfected ventral horn (orange arrows). Surprisingly, activation of D(Gi) in astrocytes did not affect the accumulation of misfolded SOD1 in nearby MN (743 ± 222 versus 730 ± 130 a.u., respectively, *P* = 0.54; Fig 10H and I) nor p62^+^ aggregates (4.3% ± 37.2% versus 4.7% ± 36.6% of aggregate burden/total cell body in the infected versus noninfected ventral horn; *P* = 0.60; Fig 10J and K) or KDEL levels (1,343 ± 433 versus 1,437 ± 343 a.u., infected versus noninfected ventral horn; *P* = 0.08; Fig 10L and M). We further extended the investigation of D(Gi) by considering the accumulation of the autophagy marker LC3A. In this case too, D(Gi) did not improve the accumulation of LC3A buildup in MNs (1,475 ± 232 versus 1,431 ± 296 a.u., respectively; Fig 10N and O). Whereas activation of astrocytic D(Gq) drives a generalized amelioration of the disease pathways (MN disease markers and BSCB integrity), stimulation of D(Gi) in astrocytes produces a dissociation between restored BSCB integrity and unmodified disease markers in MN. The D(Gi) findings imply that at this stage, BSCB impairment has limited effect on disease pathways because the BSCB can be restored without impacting ALS pathobiochemistry.

### Restoration of BSCB by astrocytic D(Gi) signaling is independent of MN firing

**Figure 11.**
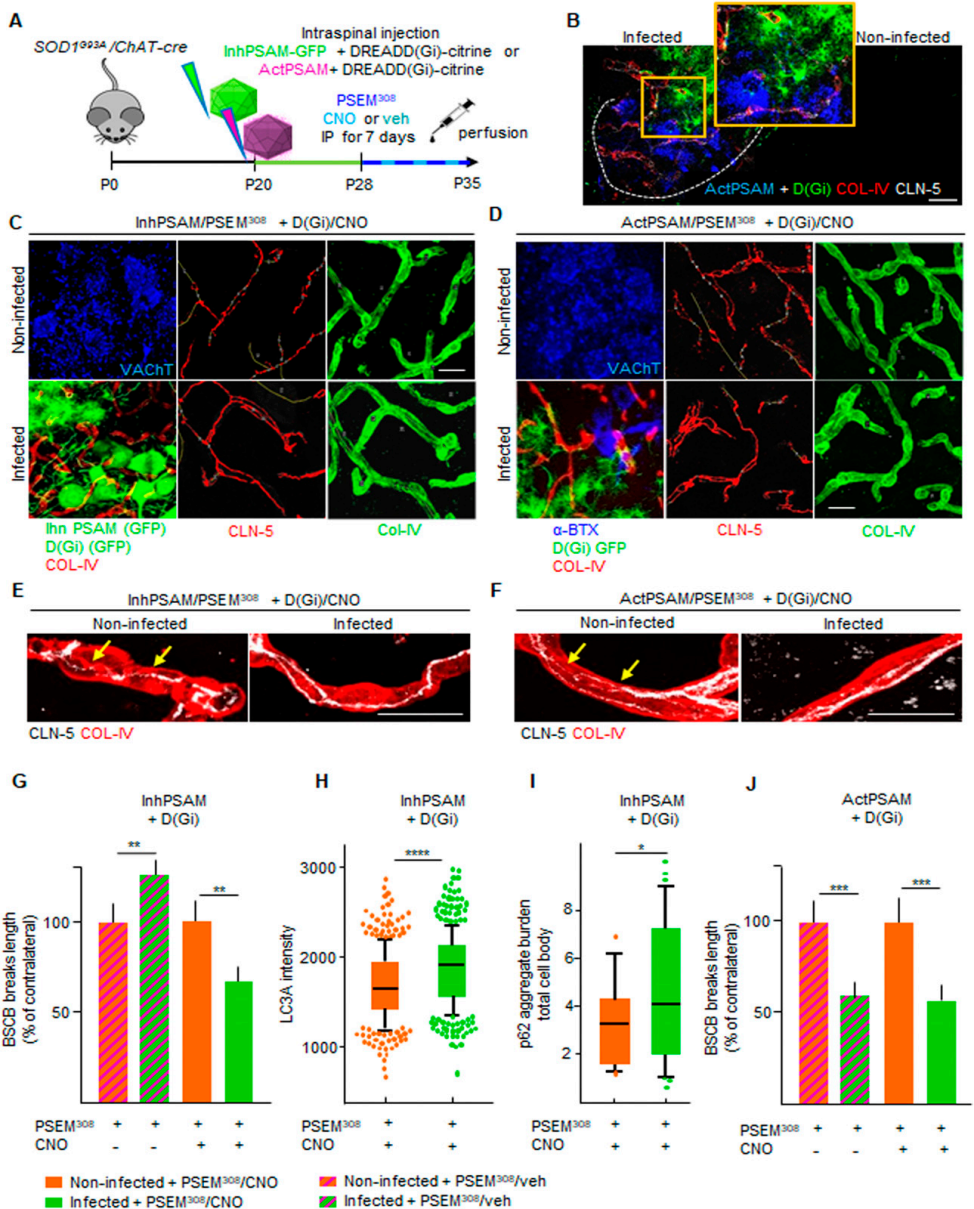
Blood–spinal cord barrier (BSCB) restoration by astrocytic D(Gi) signaling is independent of MN excitation. **(A)** Experimental design for the intraspinal injection of AAV9(hSyn::DIO-inhPSAM or -actPSAM) in combination with AAV8(GFAP::D(Gi)-Citrine) for the multiplexed chemogenetic experiments in the *SOD1*^*G93A*^*/ChAT-cre* mice at P20 and treated with the effector PSEM^308^ and the agonist CNO or vehicle from P28 until P35. **(B)** Representative picture of intraspinal injection of AAV9 encoding actPSAM, highly expressed in MNs (blue), in combination with injection of AAV8 D(Gi), highly specific for astrocytes (green) in the infected ventral horn in contrast to no expression pattern in the uninjected ventral horn. The dotted line depicts the boundary of gray and white matter. The insert shows a high magnification of infected MNs and astrocytes in the injected ventral horn. Scale bar: 50 μm. **(C)** Representative panel displaying immunostaining for the multiplexed chemogenetic experiments in which MN firing inhibition via inhPSAM/PSEM^308^ (infected MNs in green) combined with astrocytic Gi activation diminished the breaks (yellow lines) in CLN-5 ribbon (red) in the infected ventral horn compared with the uninfected contralateral ventral horn. **(D)** Panel showing immunostaining for the multiplexed chemogenetic experiment in which MN firing enhancement via actPSAM/PSEM^308^ (infected MNs detected by α-BXT in blue) combined with astrocytic Gi activation (astrocytes identified by GFP in green) further decreases breaks (yellow lines) along CLN-5 ribbon (red). MNs in the noninfected ventral horn are identified by VAChT immunostaining, whereas vessels are labeled with COL-IV (red in the combined picture and green in the single picture). **(D, E, F)** High-magnification view of CLN-5 distribution (white) along the single capillaries (red) in the multiplexed chemogenetics (E) inhPSAM/PSEM^308^ + D(Gi)/CNO and in (D) actPSAM/PSEM^308^ + D(Gi)/CNO. **(D, E)** Yellow arrows indicate the discontinuity of CLN-5 ribbon (white) in the noninfected ventral horn in contrast to a more homogeneous distribution in the infected ventral horn of (E, D) both experiments. Scale bar: 10 μm. **(G)** Quantification of BSCB disruption expressed as % of breaks of the contralateral uninfected ventral horn after multiplexed chemogenetic experiments with inhPSAM/PSEM^308^ + D(Gi)/CNO (CNO +) or with inhPSAM/PSEM^308^ + D(Gi)/veh (CNO − [column with milled pattern]). **(H, I)** Quantification of MN (H) LC3A intensity and (I) p62 aggregates per cell body in the double-infected (inhPSAM/PSEM^308^ + D(Gi)/CNO) ventral horn compared with the uninfected ventral horn. **(J)** Quantification of BSCB disruption expressed as % of breaks of the contralateral uninfected ventral horn after multiplexed chemogenetic experiments with actPSAM/PSEM^308^ + D(Gi)/CNO (CNO +) or with actPSAM/PSEM^308^ + D(Gi)/veh (CNO − [column with milled pattern]). **(H, I)** The quantifications of MN markers in (H, I) are represented by the box-and-whisker plot; 10–90 percentile is considered; N = 4–6 mice per group of experiments. Scale bars: 20 μm. Data information: in (G, H, I, J), data are presented as means ± SD. *P < 0.1, **P < 0.01, ***P < 0.001, ****P < 0.0001 (unpaired *t* test).

We then focused on D(Gi), considering that its activation dissociates the effect on BSCB integrality from MN disease markers, which could potentially shed light on the role of BSCB disruption on the pathogenesis of MN disease. We therefore investigated the mechanism involved in astrocytic D(Gi)-mediated restoration of BSCB. Because increasing MN firing was sufficient to improve BSCB integrity, we explored whether astrocytic Gi would prove beneficial by modulating MN activity, for example, by inducing the release of excitatory neurotransmitters (Durkee et al, 2019). In this case, we reasoned that any beneficial effect of D(Gi) on BSCB would be significantly decreased by the concomitant inactivation of MN firing. We arranged a multiplexed chemogenetic strategy to inactivate MN firing using the inhPSAM/PSEM^308^, while simultaneously triggering Gi signaling in astrocytes by D(Gi) (Fig 11A and B). To this end, intraspinal injections were performed in *SOD1*^*G93A*^*/ChAT-Cre* mice with AAV9 encoding for hSyn::DIO-inhPSAM (expression restricted to MN) and with AAV8 encoding GFAP::D(Gi) (expression restricted to astrocytes). After 10 d, we administered to the animals both CNO and PSEM^308^ (or vehicle + PSEM^308^) for a further 7 d (Fig 11B). Inactivation of MN alone resulted in the anticipated increased disruption of the BSCB (129.8% ± 8.7% of the contralateral noninfected ventral horn; *P* < 0.01; Fig 11C, E, and G). However, the activation of D(Gi) in the presence of MN inactivation still resulted in a substantial improvement of BSCB integrity (68.6% ± 10.7% of the contralateral uninfected ventral horn; *P* < 0.01; Fig 11C, E, and G). Nevertheless, MN firing inactivation by inhPSAM/PSEM^308^, even when co-applied with astrocytic D(Gi) activation, caused increased levels of LC3A (1,840 ± 396 versus 1,693 ± 395 a.u., respectively, in the co-injected ventral horns versus contralateral uninjected horns; *P* < 0.0001; Fig 11H) and a higher burden of p62^+^ inclusions (4.6% ± 3.1% versus 3.2% ± .1.8% of aggregate burden/total cell body, coinfected versus contralateral; *P* < 0.1; Fig 11I), confirming the effective target engagement of inhPSAM/PSEM^308^ (Saxena et al, 2013) and further demonstrating that BSCB integrity, and disease marker burden in MN can be dissociated. We further explored whether D(Gi) activation would confer an additional beneficial effect when applied together with increased MN firing (i.e., if the two beneficial effects are additive or not). We compared the CLN-5 ribbon integrity in spinal cord sections in which both MN firing (actPSAM/PSEM^308^) and astrocytic D(Gi) were activated. Notably, single chemogenetic and multiplexed chemogenetic samples displayed the same degree of BSCB restoration, (actPSAM/PSEM^308^ + D(Gi)/CNO: 60.9% ± 6.7% of contralateral noninfected versus 59.2% ± 13.1% in actPSAM/PSEM^308^ + D(Gi)/veh; *P* > 0.05; Fig 11D, F, and J). Driving MN excitation by actPSAM/PSEM^308^ therefore not only mimicked but also truly occluded the effect of astrocytic Gi signaling.

Taken together, the double-chemogenetic experiments imply that the consequences of D(Gi) on BSCB are independent of MN firing, although they mimic the effects of MN activity stimulation, suggesting that astrocytic Gi signaling may be located downstream of MN excitation.

### Astrocytic Wnt7a and Wnt5a levels are enhanced by Gi signaling in astrocytes and suppressed by MN inactivation, in correlation with BSCB modifications

**Figure 12.**
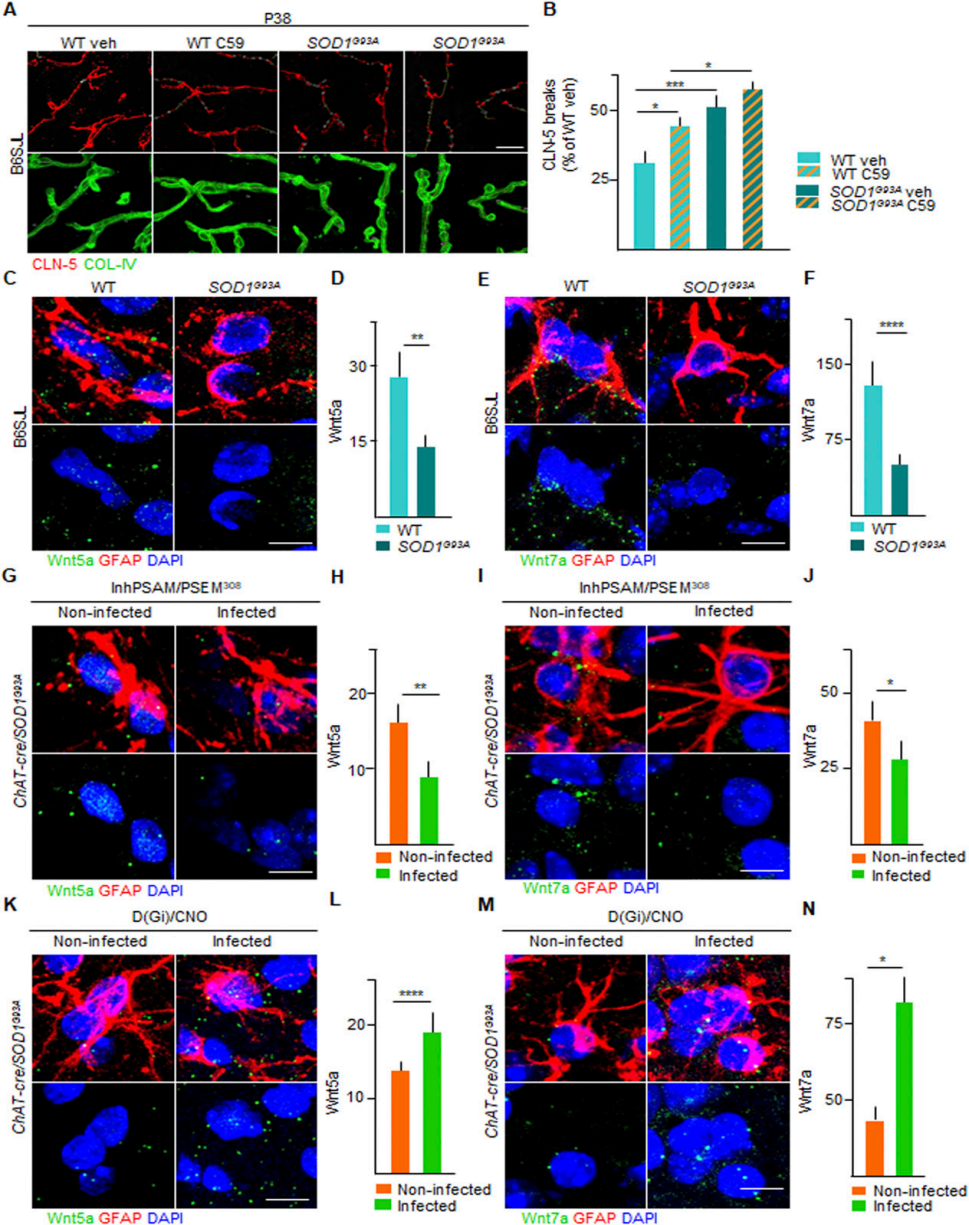
Astrocytic Wnt5a and Wnt7a mRNA up-regulation correlates with blood–spinal cord barrier restoration in amyotrophic lateral sclerosis under chemogenetic activation of astrocytic Gi signaling. **(A)** Representative panel showing immunostaining for CLN-5 (red) and COL-IV (green) in WT and *SOD1*^*G93A*^ mice at P38 treated with vehicle or porcupine (PORCN) inhibitor C-59. Breaks of CLN-5 ribbon (red) along the vessels (green) are marked with yellow lines. Scale bars: 20 μm. **(B)** Quantification of CLN-5 breaks length (expressed as % of WT [treated with vehicle] breaks) in WT and *SOD1*^*G93A*^ mice treated with vehicle or C-59 (column with milled pattern). Data are from N = 3. **(C, E)** Representative picture of detection by in situ *hybridization* of (C) Wnt5a and (E) Wnt7a mRNA (green dots) in astrocytes, identified by glial fibrillary acidic protein immunofluorescence staining (red) in WT and *SOD1*^*G93A*^ mice. Nuclei are depicted with DAPI (blue). **(D, F)** Quantification of mRNA levels of (D) Wnt5a and (F) Wnt7a in WT and *SOD1*^*G93A*^ mice, expressed as the amount of dots. **(G, I)** Representative panel showing detection by in situ *hybridization* of (G) Wnt5a and (I) Wnt7a mRNA (green dots) in astrocytes in the inhPSAM/PSEM^308^ chemogenetic experiment. Astrocytes are identified by glial fibrillary acidic protein immunofluorescence staining (red). Nuclei are depicted with DAPI (blue). **(H, J)** Quantification of (H) Wnt5a and (J) Wnt7a mRNA amount in infected and uninfected contralateral ventral horns of the spinal cord of *SOD1*^*G93A*^*/ChAT-cre* mice injected with AAV9 inhPSAM/PSEM^308^. Data are from N = 4. **(K, M)** Representative panel showing detection by in situ *hybridization* of (K) Wnt5a and (M) Wnt7a mRNA (green dots) in astrocytes in the D(Gi)/CNO chemogenetic experiment. Nuclei are depicted with DAPI (blue). **(L, N)** Quantification of (L) Wnt5a and (N) Wnt7a mRNA amount in infected and uninfected contralateral ventral horns of the spinal cord of *SOD1*^*G93A*^*/ChAT-cre* mice subjected to D(Gi)/CNO chemogenetic treatment. Data are from N = 4. Scale bars: 5 μm. Data information: in (B, D, F, H, J, L, N), data are presented as means ± SD. **(B, D, F, H, J, L, N)** *P < 0.1, **P < 0.01, ***P < 0.001, ****P < 0.0001 (one-way ANOVA with Bonferroni correction for multiple comparisons [B] and unpaired *t* test [D, F, H, J, L, N]).

We sought to identify the mediator(s) involved in the sealing of the BSCB induced by the activation of Gi signaling in astrocytes. We focused on the Wnt family because these mediators are necessary in the establishment of the blood-brain-barrier (BBB) during development (Liebner et al, 2008), as well as in its maintenance in adulthood (Tran et al, 2016; LeBlanc et al, 2019). First, we explored if the blockade of Wnt signaling modified the integrity of the BSCB in WT and *SOD1*^*G93A*^ mice. The animals were treated for 14 d either with vehicle or with the porcupine (PORCN) inhibitor (which blocks Wnt acylation and release) C59 (Proffitt et al, 2013; Torres et al, 2019), starting from day P20. The administration of the Wnt antagonist C59 caused a small disruption of the BSCB in the WT mice (124.7% ± 16.4% of WT control mice treated with vehicle) and a substantial worsening of the BSCB integrity in the *SOD1*^*G93A*^ mice (157.2% ± 17.7% of WT veh; *P* < 0.0001; Fig 12A and B), indicating that Wnt proteins are involved in balancing the BSCB integrity in the ALS mice.

Next, we focused on Wnt5a, the most abundant Wnt family member in the spinal cord (Ouali Alami et al, 2018), recognised as sufficient for inducing a BBB-like phenotype in endothelial cells (Artus et al, 2014) and on Wnt7a, a critical mediator in the establishment and maintenance of the BBB phenotype (Cho et al, 2017; Wang et al, 2018). We exploited single-molecule mRNA in situ hybridization (coupled to immunostaining for the identification of cellular subpopulations) to quantify Wnt5a and Wnt7a in spinal cord astrocytes (identified by GFAP staining). Both Wnt5a and Wnt7a mRNA molecules were readily detected in GFAP^+^ astrocytes in the WT and *SOD1*^*G93A*^ spinal cord sections (Fig 12C–F). Notably, both astrocytic Wnt5a mRNA molecules (58.6% ± 12.3% of WT; *P* < 0.01; Fig 12C and D) and Wnt7a mRNA molecules (28.6% ± 11.9% of WT; *P* < 0.0001; Fig 12E and F) were significantly decreased in the *SOD1*^*G93A*^ mice compared with WT controls.

We then explored if Wnt5a and Wnt7a levels could be modulated by chemogenetic manipulations in correlation with the integrity of the BSCB. We determined that chemogenetic inactivation of MN firing by inhPSAM/PSEM^308^ caused a significant additional decrease in Wnt5a mRNA (40.5% ± 13.1% of contralateral; *P* < 0.0001; Fig 12G and H) and in the Wnt7a mRNA molecules (71.9% ± 11.6% of the uninfected contralateral horn; *P* < 0.01; Fig 12I and J) in the astrocytes of the infected ventral horn compared with the contralateral side. These data suggest that disruption of the BSCB driven by MN inactivation is associated with the reduced expression of Wnt5a and Wnt7a in astrocytes. We tested this hypothesis by assessing the impact of astrocytic Wnt5a and Wnt7a levels on D(Gi) activation in astrocytes. We obtained a strong increase in Wnt5a (180.8% ± 33.3% of contralateral; *P* < 0.01; Fig 12K and L) and Wnt7a mRNA molecules (219.4% ± 6.4%; *P* < 0.01; Fig 12M and N) in the infected astrocytes compared with the uninfected astrocytes in the contralateral side. Wnt5a and Wnt7a levels were therefore highly correlated with the integrity of BSCB during the pharmacological inhibition of Wnt signaling, as well as with an increase in MN firing and stimulation of astrocytic Gi signaling.

### Activation of astrocytic Gi signaling at later stages ameliorates both BSCB disruption and disease burden

**Figure 13.**
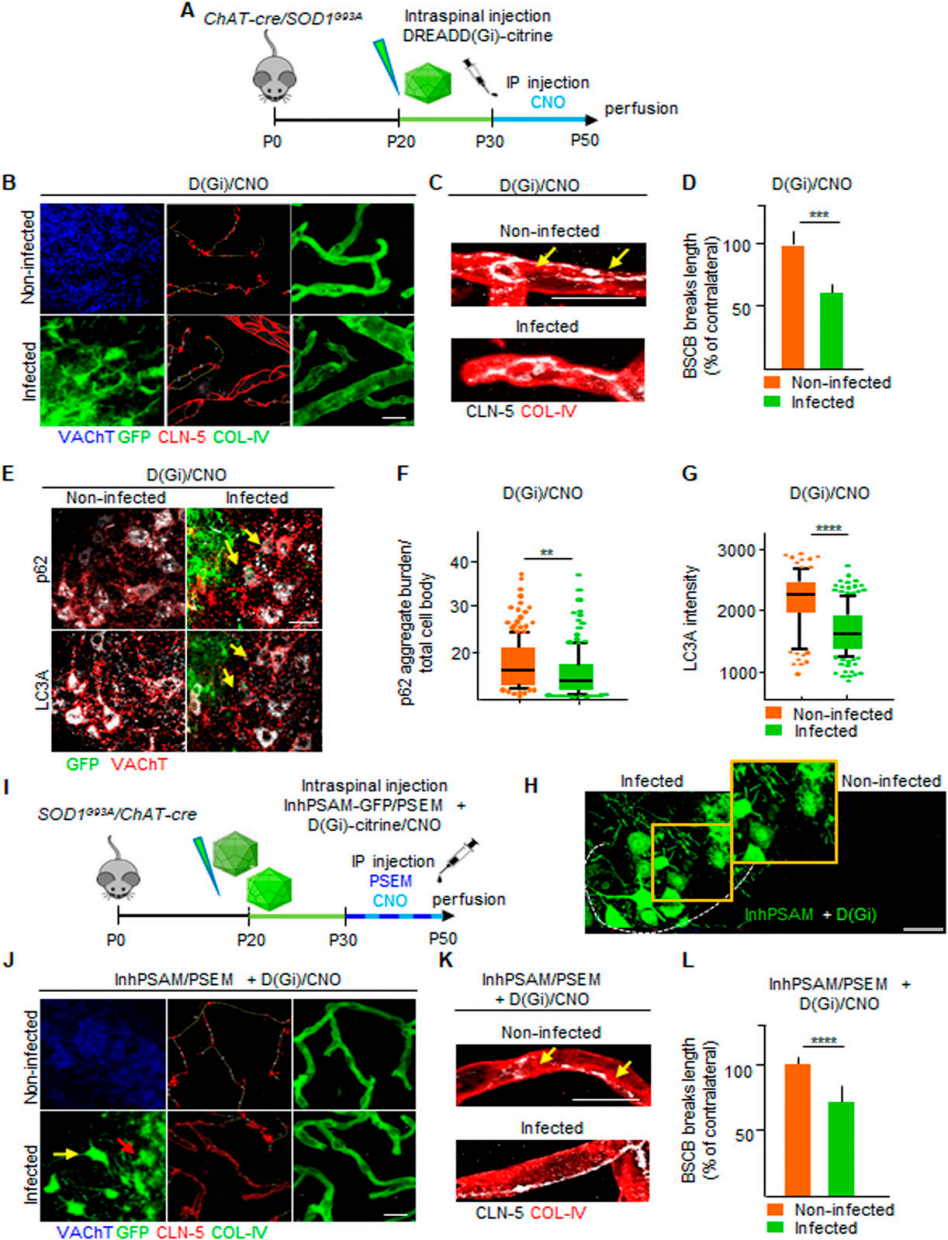
Chemogenetic activation of astrocytic D(Gi) at later amyotrophic lateral sclerosis stage ameliorates microvessel integrity and MN disease markers. **(A)** Experimental design for the injection of AAV8 encoding D(Gi) in *SOD1*^*G93A*^*/ChAT-cre* mice, at P20 and treated with the agonist CNO from P30 until P50. **(B)** Representative panel displaying the effect of the prolonged and late activation of astrocytic (DGi) on CLN-5 distribution (red) along the vessels (green). MNs are identified by VAChT immunostaining (blue), and infected astrocytes are stained with GFP (green). CLN-5 breaks are depicted in yellow lines. **(C)** High magnification of CLN-5 organization (white) along COL-IV^+^ vessels (red). Yellow arrows indicate CLN-5 interruptions along the ribbon in the noninfected ventral horn of *SOD1*^*G93A*^*/ChAT-cre* mice subjected to D(Gi) late and prolonged activation. Scale bars: 10 μm. **(D)** Quantification of blood–spinal cord barrier (BSCB) disruption, expressed as % of contralateral breaks length. **(E)** Representative pictures showing MN markers p62 and LC3A immunofluorescence staining (gray) in infected and uninfected contralateral ventral horn of *SOD1*^*G93A*^*/ChAT-cre* spinal cord sections. MNs are detected by VAChT immunostaining (red) and infected astrocytes by GFP (green). **(F, G)** Quantification of (F) p62 aggregates per cell body and (G) LC3A intensity in MN surrounding infected astrocytes and in MN located in the uninfected contralateral ventral horn. The quantifications are represented by the box-and-whisker plot; 10–90 percentile is considered. Data are from N = 3 mice. **(I)** Experimental design for multiplexed chemogenetic injection of AAV9 encoding inhPSAM + D(Gi) in *SOD1*^*G93A*^*/ChAT-cre* mice, at P20 and treated with respective ligands PSEM^308^ and CNO from P30 until P50. **(H)** Expression pattern of MNs activated by inhPSAM (green) and astrocytes activated by D(Gi). Dotted lines delineate the contour of the gray and white matter in the ventral horns of the spinal cord. The insert highlights infected MNs (green) surrounded by infected astrocytes (green). Scale bar: 50 μm. **(J)** Representative panel displaying the effect of the prolonged and late activation of multiplexed chemogenetic inhPSAM/PSEM^308^ + D(Gi)/CNO experiments on the BSCB grade of disruption in *SOD1*^*G93A*^*/ChAT-cre* mice. MNs in the noninfected horn are identified by VAChT immunostaining (blue), whereas infected MNs and astrocytes are stained with GFP (green). CLN-5 breaks are depicted in yellow lines along the ribbon (red). **(K)** High magnification of CLN-5 organization (white) along COL-IV^+^ vessels (red). Yellow arrows indicate CLN-5 interruptions along the ribbon in the noninfected ventral horn of *SOD1*^*G93A*^*/ChAT-cre* mice subjected to the late and prolonged multiplexed chemogenetic experiments. Scale bars: 10 μm. **(L)** Quantification of breaks length in the BSCB of *SOD1*^*G93A*^*/ChAT-cre* mice subjected to the late and prolonged multiplexed chemogenetic experiments, expressed as % of contralateral. Data are from N = 3 mice. Scale bars: 20 μm. Data information: in (D, F, G, L), data are presented as means ± SD. **P < 0.01, ***P < 0.001, ****P < 0.0001 (unpaired *t* test).

Because ALS pathogenic processes are heterogeneous and evolve over time (Ouali Alami et al, 2018), we investigated whether prolonged D(Gi) activation would enable the restoration of the BSCB in correspondence to the first wave of MN denervation (taking place at about P50; [Pun et al, 2006]). *SOD1*^*G93A*^ mice were injected with AAV8(GFAP::D(Gi)-Citrine) at P20 and treated with a single injection CNO daily for 20 d, from P30 until P50 (Fig 13A). We observed that prolonged activation of astrocytic D(Gi) resulted in a significant decrease in the breakdown of the BSCB (CLN-5 breaks length, 59.9% ± 8.0% in D(Gi) versus uninfected contralateral controls; *P* < 0.001; Fig 13B and D). Surprisingly, the prolonged activation of astrocytic D(Gi) affected the burden of MN disease markers. The MN nearby D(Gi)-expressing astrocytes displayed a reduced burden of p62^+^ aggregates (11.1% ± 10.1% versus 20.4% ± 54.9% in uninfected contralateral MN, aggregate burden/total cell body; *P* < 0.01; Fig 13E and F) and decreased levels of LC3A (1,657 ± 386 a.u. versus 2,180 ± 485 a.u., respectively; *P* < 0.0001; Fig 13E and G).

At the same time, the amount of BSCB restoration remained substantial even upon simultaneous MN inactivation by inhPSAM (CLN-5 breaks length, 72.7% ± 8% of uninfected contralateral side; *P* < 0.0001; Fig 13H–L), confirming the independence of the astrocytic D(Gi) from MN excitability effects.

In conclusion, although BSCB restoration, driven by Gi signaling in astrocytes at early stages of the disease, was not associated with any effect on disease markers, it could be linked to neuroprotective effects at later phases of ALS pathology.

## Discussion

In the present work, we have showed that (i) BSCB structural and functional disruption is detected in four ALS mouse lines with unrelated mutations and different degrees of MN loss; (ii) the disruption of the BSCB takes place before any MN loss (in at least two lines), and it is restored by enhancing MN excitability; and (iii) the activation of Gi signaling in astrocytes reverts the disruption of the BSCB by increasing Wnt5a/Wnt7a expression but without impacting the burden of disease markers in MN and independently of MN firing. Overall, these data suggest first that in healthy conditions, MN excitability controls BSCB integrity through astrocytes and second that in ALS, the loss of MN excitability and firing leads (possibly through the decrease in signaling driven by the Gi-coupled receptor) to the down-regulation of Wnt5a/7a in astrocytes, which in turn provokes the opening of the BSCB. Whereas at the very initial stages, the restoration of BSCB integrity does not affect MN pathobiochemistry, the opposite is true later on: the disruption of the BSCB is an event secondary to MN dysfunction, which then feeds back on MN and aggravates the ongoing pathogenic process.

Although the disruption of the BSCB in ALS has been previously reported (Garbuzova-Davis et al, 2007a, 2007b; Zhong et al, 2008), the nature of the primum movens has remained debated: Is BSCB impairment driven by MN dysfunction or is it a MN-independent event? Some (controversial) evidence has pointed toward an MN-independent origin: Endothelial cells expressing mutant *SOD1* in vitro display a cell-autonomous disruption of TJs because of the misfolded protein itself (Meister et al, 2015). At the same time, endothelial cell–selective excision of the mutant *SOD1* transgene does not prevent the breakdown of the BSCB nor affect the survival of the transgenic mice (Zhong et al, 2009). The loss of pericytes observed in spinal cord samples from ALS patients has been hypothesized to be at the origin of BSCB disruption (Winkler et al, 2013). However, increased PDGF-C signaling appears to be associated with BSCB disruption in ALS (Liebner et al, 2008), even if PDGFR-α signaling is actually necessary for the survival of these cells (Kisler et al, 2017). Recently, microglial activation, alone or together with peripheral immune cells contribution, has been hypothesized to be involved in the opening of the BSCB (Puentes et al, 2016; Frakes et al, 2017; Epperly et al, 2019). We have demonstrated that chemogenetic control of MN firing can bidirectionally modulate BSCB integrity: Increased firing reduces BSCB disruption, whereas neuronal inactivation worsens it. This pattern corresponds to what has been observed for other disease markers such as misfolded SOD1 accumulation, ER stress, and unfolded protein response (Saxena et al, 2013; Bączyk et al, 2020). Even if other cell types play contributing roles, the impairment of the BSCB is thus the manifestation of a pathogenic process, primarily originating in MN. Notably, BSCB impairment is detected in ALS mouse models before any MN loss (such as in the *FUS*^Δ*NLS*^ and *SOD1*^*G93A*^) but at stages when hypoexcitability of vulnerable MN is already detectable (Martinez-Silva et al, 2018). Thereafter, the disruption of the BSCB may be a consequence of early MN dysfunction.

How is this dysfunction translated into a vascular phenotype? Considering that astrocytes are sensitive to neuronal activity (through several glutamatergic, GABAergic, cholinergic, and purinergic GPCR; Kofuji & Araque, 2020) and, at the same time, they are key organizers of the BSCB (Abbott et al, 2006), these cells become prime candidates for investigation. We exploited DREADDs with astrocyte-selective promoters to obtain a direct cell- and time-specific modulation of GPCR signaling (Orr et al, 2015; Bang et al, 2016; Adamsky et al, 2018; Jones et al, 2018; Nagai et al, 2019). We demonstrated that D(Gq) and D(Gi) can induce the restoration of BSCB integrity, although D(Gq) itself appears to be beneficial toward all disease readouts, suggesting that it may restore the BSCB by ameliorating MN firing, possibly upon triggering gliotransmitter release (Durkee et al, 2019). Critically, D(Gi) signaling acts directly on the BSCB, independently of MN firing, as we have shown in our multiplexed chemogenetic strategy.

How does astrocytic D(Gi) induce the restoration of the BSCB? We elected to investigate the role of Wnt proteins, in particular Wnt5a and Wnt7a. Wnt family members are involved in the establishment of the BBB during development (Liebner et al, 2008; Stenman et al, 2008) and contribute to its maintenance in the adult brain (Artus et al, 2014; Tran et al, 2016). Low Wnt/β-catenin signaling actually characterizes the site in the brain with the lowest integrity of the BBB and the highest permeability (Benz et al, 2019). Among Wnt family members, Wnt7a appears to be critical during development (Daneman et al, 2009) and maintains the integrity of the BBB through Gpr124, during a stroke or in glioblastoma (Chang et al, 2017), as well as in homeostatic conditions. Nevertheless, Wnt5a is sufficient to induce a BBB phenotype in endothelial cells in vitro (Artus et al, 2014), and it is among the most strongly expressed Wnt members in astrocytes (Ouali Alami et al, 2018). We have demonstrated that Wnt5 expression in astrocytes is reduced early on in *SOD1*^*G93A*^ mice, and the suppression of Wnt signaling by the porcupine inhibitor C59 further worsens BSCB disruption. Moreover, the astrocytic Wnt5a mRNA and Wnt7a mRNA levels bidirectionally correlate with the integrity of the BSCB, being down-regulated upon inactivation of MN firing (worsening of the BSCB integrity) and up-regulated by D(Gi) activation in astrocytes (ameliorating the BSCB integrity). During increased availability of Wnt5a and Wnt7a, it is believed that the activation of the β-catenin signaling cascade in endothelial cells is a key event in inducing and maintaining BSCB specializations. Canonical Wnt signaling through β-catenin is sufficient to induce a BBB-like phenotype in cultured endothelial cells (Laksitorini et al, 2019), and it is critically involved in establishing the BBB phenotype in vivo (Liebner et al, 2008; Chang et al, 2017; Cho et al, 2017; Wang et al, 2018), whereas reduced Wnt/β-catenin signaling spatially corresponds to sites of high BBB permeability in the brain (Benz et al, 2019). Moreover, noncanonical Wnt signaling may contribute to the establishment of the BBB phenotype (Pinzón-Daza et al, 2014). Sox17 has been identified as a major target of the β-catenin pathway in endothelial cells (Corada et al, 2019), together with Lef1 and Ets1 (Roudnicky et al, 2020). These Wnt-regulated transcription factors up-regulate the transcription of several junctional proteins, including VE-cadherin and claudin-5 (Roudnicky et al, 2020), possibly involved in the repair of the BSCB. Furthermore, claudin-5 is dynamically internalized and stored in intracellular vesicles upon inflammatory stimuli and trafficked back to the cell surface when the inflammatory signaling is subsided (Stamatovic et al, 2003, 2006, 2009). It is therefore conceivable that transcriptional mechanisms and trafficking may be responsible for Wnt5a/7a-driven BSCB restoration.

In principle, it is possible that additional mediators may contribute to the D(Gi) effect (e.g., GDNF; Igarashi et al, 1999). One could also hypothesize that the up-regulation of Wnt5a/7a induced by D(Gi) may affect other nonneuronal cells (e.g., microglia) and only indirectly play a role in the integrity of the BSCB. Although this model is theoretically possible and worth further investigation, the direct effects of Wnt5a/7a on BSCB integrity are well established (Liebner et al, 2008; Stenman et al, 2008; Artus et al, 2014; Tran et al, 2016; Cho et al, 2017; Wang et al, 2018; Benz et al, 2019; Laksitorini et al, 2019; LeBlanc et al, 2019). Because microglia do respond to astrocytic Wnt (Ouali Alami et al, 2018), Wnt signaling is thought to cause microglial proliferation and substantial pro-inflammatory activation (Halleskog et al., 2012, 2011), although the ultimate outcome may depend on the context (Halleskog and Schulte, 2013). Putative indirect actions of astrocytic D(Gi) remain to be further investigated.

**Figure S5.**
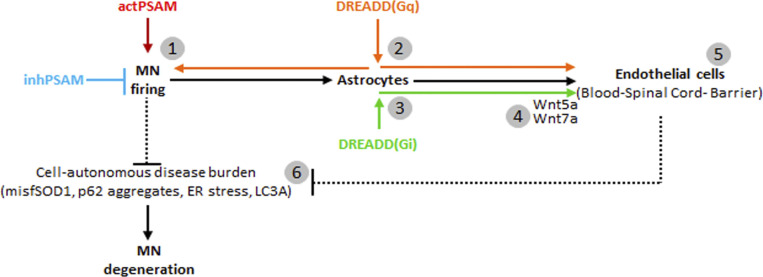
Interpretative model of the chemogenetic dissection of neuro–glio–vascular interactions in amyotrophic lateral sclerosis. In amyotrophic lateral sclerosis mice, enhancement or depression of MN excitability using actPSAM (red) or inhPSAM (blue) **(1)** is sufficient to reduce or increase, respectively, the disruption of the blood–spinal cord barrier (BSCB). The interaction between MN firing **(1)** and BSCB integrity **(5)** is hypothesized to be mediated by astrocytes **(2)** possibly through G protein–coupled receptor. Nevertheless, activation of G protein–coupled receptor signaling in astrocytes is sufficient to restore BSCB integrity: Whereas astrocytic Ca^2+^-PKC-coupled DREADD(Gq) (orange) restores BSCB together with MN disease burden **(2)**, the DREADD(Gi) (green) restores BSCB independently of MN firing **(3)** by up-regulating the expression of Wnt7a and Wnt5a **(4)**. Whereas, at the very early stages of the pathogenic cascade, the restoration of the BSCB **(5)** does not affect disease manifestation, the opposite is true at later stages of disease progression, when restoration of the BSCB helps to reduce the disease burden in MNs **(6)**.

In our current interpretative model (Fig S5), MN activity is hypothesized to be linked to BSCB integrity via astrocytes and astrocyte-secreted Wnt5a/7a. Nevertheless, to date, virtually nothing is known about the MN-originated signals that modulate Wnt5a/7a in adult astrocytes. However, one can speculate about the role of astrocytic GPCRs involved in monitoring neuronal and synaptic activities (e.g., GABA, glutamate, ATP, and acetylcholine receptors are expressed by astrocytes; Kofuji & Araque, 2020). Insufficient engagement of these receptors due to MN hypoexcitability and reduced synaptic excitation (Martinez-Silva et al, 2018; Bączyk et al, 2020) may also decrease Wnt5a/7a in astrocytes. One could further speculate that MN-initiated signaling might converge on NF-kB because overactivation of IKK-β in astrocytes is sufficient to increase Wnt5a expression (Ouali Alami et al, 2018).

The ability to control BSCB integrity in vivo enables us to address a critical issue in the vascular biology of ALS: Is the disruption of the BSCB detrimental to the MN disease process? Previous evidence suggests that closure of the BSCB by APC administration is associated with better outcomes; however, this effect was linked in reality to the decrease in the expression of the mutant *SOD1* gene itself induced by APC (Zhong et al, 2009). Likewise, the worsening of disease progression observed upon warfarin administration has been attributed to the substantial disruption of the BSCB, the extravasation of erythrocytes, and iron-triggered radical oxygen toxicity (Winkler et al, 2014). Nevertheless, this interpretation is complicated by the unclear target selectivity of warfarin itself and by the lack of an identified cellular target. There is further proof that even the substantial disruption of the BSCB that follows NF-κB activation in astrocytes is not necessarily associated with a worsening of the disease progression (Ouali Alami et al, 2018). Furthermore, mutant *SOD1* mice lacking aquaporin-4 display an intact BSCB but do not have a better disease course and survival (Watanabe-Matsumoto et al, 2018). Our data show that the activation of D(Gi) in astrocytes restores BSCB integrity without ameliorating the burden of multiple disease markers in MN (effectively dissociating the BSCB from other disease manifestations). This suggests that at the initial stages of the disease, BSCB impairment does not necessarily carry pathogenic consequences.

At the same time, our findings are compatible with a possible pathogenic role of BSCB impairment later in disease progression because at this stage, Gi signaling in astrocytes restores the BSCB (once again independently of MN firing), while it reduces the burden of disease markers. However, some limitations apply to later-stage results: Gi signaling activates a number of biological processes in astrocytes, whereas the role of astrocytes may change over time (Ouali Alami et al, 2018). Although it is not possible to draw an unequivocal causal link between BSCB restoration and disease burden, it is still plausible that at this stage, BSCB restoration might be the consequence of the decrease in disease burden.

In conclusion, we demonstrated that BSCB impairment originates as a consequence of early MN dysfunction and can be dissociated from MN disease burden through the selective activation of Gi signaling in astrocytes and the consequent induction of Wnt proteins (in particular Wnt5a/7a). This discovery implies that BSCB disruption does not have a pathogenic role at the early stages of the disease. However, prolonged Gi signaling in astrocytes beneficially affects BSCB function at later stages of the disease by decreasing or delaying MN disease burden.

## Materials and Methods

### Experimental animals

The experiments were approved by the Tierforschungszentrum-Ulm and by the Regierungspräsidium Tübingen (Germany) under license no. 1404. The following strains of transgenic mice were used: B6SJL-Tg(SOD1*G93A)1Gur/J (high-copy, henceforth *SOD1*^*G93A*^) and *B6.Cg-Tg(SOD1)2Gur/J* (henceforth WT-SOD1) mice were obtained from Jackson laboratories; *B6N.129S6(B6)-Chattm2(cre)Lowl/J* (henceforth *ChAT-Cre*) were a kind gift from Pico Caroni (FMI); *C57BL/6NCrl-FUS(*Δ*NLS)* (henceforth *FUS*^*(*Δ*NLS+/−)*^) were obtained from Luc Dupuis (INSERM; [Scekic-Zahirovic et al, 2016]) and bred locally; *B6SJL/F1-TDP-43(G298S)* (henceforth *TDP-43*^*(G298S)*^) were a kind gift from Phil Wong (Johns Hopkins School of Medicine [Wiesner et al, 2018]); and *B6.129P2-Tbk1tm1Aki* (henceforth *Tbk1*^*+/−*^) were a kind gift from Jochen Weishaupt (Ulm University, Ulm; [Brenner et al, 2019]). To generate *SOD1*^*G93A*^*/ChAT-Cre* double-transgenic mice, *SOD1*^*G93A+*^ male mice were crossed with *ChAT-Cre*^*+/+*^ female mice, and the double-positive male mice in the F1 were used for the experiments.

All experiments were carried out on male mice because of the known difference in disease progression rate between male and female mice and of the less predictable time course in female mice, starting from the age of P20. Unless otherwise specified, animals were kept in groups of 3–4 mice in a 12-h light–dark cycle and were given ad libitum access to food and water. Mice were checked daily for the appearance of symptoms. Based on power calculations (α = 80% with 5% significance two-sided) for each experimental group or time points, 6–8 animals were processed and analyzed.

### AAV vectors

The following AAV vectors were obtained from Addgene: AAV serotype 8, pAAV(8)-GFAP-GFP, pAAV(8)-GFAP-hM3D(Gq)-Citrine, pAAV(8)-GFAP-hM3D(Gs)-Citrine, and pAAV(8)-GFAP-hM4D(Gi)-Citrine. The plasmids encoding for pAAV(9)-CBA-GFP-2A-floxed-PSAM(L141F,Y115F)GlyR-WPRE and pAAV(9)-pCAG-A7-floxed-PSAM(L141F, Y115F)5HT3-WPRE were a kind gift from Scott Sternson, HHMI-Janelia (Magnus et al, 2011), and were assembled in AAV9 vectors (details of the preparation of the vectors are reported in Supplemental Data 1; Matsushita et al, 1998; Aurnhammer et al, 2011; Hussaini et al, 2013; Jungmann et al, 2017; Commisso et al, 2018).

Supplemental Data 1. AAV production, intraspinal injection, histology and immunostaining, single-molecule in situ mRNA hybridization, confocal imaging and image analysis.

### Intraspinal injection of AAV

Intraspinal injection of AAV was performed as previously reported (Saxena et al, 2013). Briefly, the spinal cord was accessed by a dorsal laminectomy performed at the T11–T13 level. Injection (1 μl) was performed using a pulled glass capillary coupled to a Picospritzer-III apparatus. Using the central dorsal artery as reference, injections were performed at the coordinates y = +0.30; z = −0.45. Further details are reported in Supplemental Data 1.

### Chemogenetics agonist administration

CNO was purchased from Tocris and was administered by i.p. injection at a dose of 5 mg/kg once daily, starting 10 d after the intraspinal injection (unless otherwise specified) for 7 consecutive days. The effector molecule PSEM^308^ was custom synthesized by Apex Scientific and was administered daily at a dose of 5 mg/kg in saline, starting from 10 d after viral injection for 7 consecutive days; because PSEM^308^ is poorly soluble in cold saline, stock solutions were pre-warmed at 40°C for 1 h before injection and used at RT.

### C59 administration

The C59 inhibitor of porcupine (PORCN) was suspended in PBS + 5% Tween 80 + 5% PEG-400 and was administered to SOD1^G93A^ and WT mice at the age of 20–22 d, via oral gavage (10 mg/kg) for 14 d. Vehicle (composed of PBS, Tween 80, and PEG-400) was administered to control SOD1^G93A^ and WT mice.

### Western blot

For the preparation of whole spinal cord protein extract, the lumbar spinal cord was dissected after mouse euthanasia by cervical dislocation and snap frozen on dry ice as previously reported (Olde Heuvel et al, 2019). Briefly, lumbar spinal cord tissue was dissected and homogenized in a complete radioimmunoprecipitation assay (RIPA) buffer, containing protease and phosphatase inhibitors. Samples were then subjected to SDS/PAGE gel electrophoresis, transferred to a polyvinylidene difluoride (PVDF) or nitrocellulose membrane, and subsequently incubated overnight at 4°C with the following primary antibodies: total anti-TDP 43 rabbit (10782-2-AP; 1:2,000 buffered in 1% BSA in PBS containing 0.05% Tween 20; Proteintech), anti-FUS rabbit (1:2,000 buffered in 1% BSA in PBS containing 0.05% Tween 20; Bethyl Laboratories), and anti-SOD1 rabbit (Prestige 001401; 1:1,000 buffered in 1% BSA; in PBS containing 0.05% Tween 20; Sigma-Aldrich). After washing steps in PBS enriched with 0.05% Tween 20, the membranes (nitrocellulose membrane for SOD1 and PVDF for TDP43 and FUS) were subsequently incubated for 1 h at RT with goat anti-rabbit IgG-HRP-conjugated secondary antibody (1:10,000 in PBS-Tween 20; Bio-Rad). After appropriate washing steps, the membranes were further treated with Western ECL-immunodetection buffer (Bio-Rad) and acquired using Image Lab 5.0. ThermoScientific PageRuler Plus Prestained was used as a protein ladder. Samples were corrected for background, and densitometry analysis was performed using Image Lab software5.0. To control variability in loading individual samples, the signal intensity of protein bands was normalized to housekeeping GAPDH protein.

### Histology and immunostaining

The spinal cord was prepared for histology and immunostaining as previously reported (Saxena et al, 2013; Ouali Alami et al, 2018). Briefly, after perfusion, fixation with 4% PFA, and cryoprotection in 30% sucrose, 40-μm-thick sections were subject to antigen retrieval (whenever indicated) and immunostained using a free-floating protocol. Details of the immunostaining procedure are reported in Supplemental Data 1, and a list of the antibodies and other reagents used is reported in Table S1.

Table S1 Antibodies and in-situ probes.

### Claudin-5 breaks quantification

The loss of CLN-5 ribbon is defined by a lack of CLN-5 immunostaining under specific threshold by taking as reference the capillary wall stained with collagen-IV. The length breaks are measured and traced (in yellow) with ImageJ software. The quantification is performed by relating the total length of the single breaks and the total length of the vessels, outlined by collagen-IV, and expressed as % of breaks on the total vessels. Images are acquired as single tile scans, covering the ventral horn of the spinal cord, with specific Z-stack at the confocal microscope and analyzed as collapsed pictures.

### Single-molecule in situ mRNA hybridization

Detection of mRNA in situ together with co-immunostaining was performed as previously reported (Olde Heuvel et al, 2019) and according to the manufacturer’s recommendation (Acd Bio). Details of the procedure are reported in Supplemental Data 1, and details of the probes used are reported in Table S1.

### Confocal imaging and image analysis

Confocal images were acquired as previously reported (Ouali Alami et al, 2018). Details of image acquisition are reported in Supplemental Data 1.

The quantification of disease burden markers (misfolded SOD1, LC3A, KDEL, and p62) was performed as previously reported (Saxena et al, 2013; Ouali Alami et al, 2018). Detailed procedures are reported in Supplemental Data 1.

For the quantification of the structural disruption of the BSCB, we considered a ROI in the ventral spinal cord. We traced the total length of vessels, covered by collagen-IV staining, in the ROI and the length of vascular segments in which the claudin-5 ribbon appeared disrupted or fragmented (“gaps” or “breaks,” depicted in yellow lines) and computed the ratio between the cumulative gap length and the total length of the vessels, as previously reported (Ouali Alami et al, 2018).

For the quantification of the astrocytic end-feet coverage of spinal cord vessels, blood vessels were first identified using collagen-IV staining, and a ROI corresponding to the vessels was manually traced; the surface of the vessel occupied by GFP-positive processes was then quantified, and a ratio between the GFP^+^ area and the total vessel area was considered. The same procedure was used for the quantification of AQP4 around the microvessels. For quantitative analysis, a minimum of 8–10 artifact-free sections per mouse was analyzed, and from each 16–20 vessel, stretches were taken into consideration.

### Statistical analysis

One-way ANOVA with Bonferroni correction for multiple comparisons was applied for the comparison of multiple groups. For the comparison of multiple groups with various conditions, two-way ANOVA and nonparametric statistical analysis were applied. The unpaired *t* test was used to determine if the difference between two groups is significant. Statistical analysis was performed with Prism software (GraphPad6 and 8). All values were expressed as mean ± SD unless otherwise indicated. Statistical significance was set at *P* < 0.05 before multiple comparison correction.

## Supplementary Material

Reviewer comments

## References

[bib1] Abbott NJ, Rönnbäck L, Hansson E (2006) Astrocyte–endothelial interactions at the blood–brain barrier. Nat Rev Neurosci 7: 41 10.1038/nrn1824 16371949

[bib2] Adamsky A, Kol A, Kreisel T, Doron A, Ozeri-Engelhard N, Melcer T, Refaeli R, Horn H, Regev L, Groysman M (2018) Astrocytic activation generates de novo neuronal potentiation and memory enhancement. Cell 174: 59–71.e14. 10.1016/j.cell.2018.05.002 29804835

[bib3] Artus C, Glacial F, Ganeshamoorthy K, Ziegler N, Godet M, Guilbert T, Liebner S, Couraud P-O (2014) The Wnt/planar cell polarity signaling pathway contributes to the integrity of tight junctions in brain endothelial cells. J Cereb Blood Flow Metab 34: 433–440. 10.1038/jcbfm.2013.213 24346691 PMC3948118

[bib4] Aurnhammer C, Haase M, Muether N, Hausl M, Rauschhuber C, Huber I, Nitschko H, Busch U, Sing A, Ehrhardt A (2011) Universal real-time PCR for the detection and quantification of adeno-associated virus serotype 2-derived inverted terminal repeat sequences. Hum Gene Ther B Methods 23: 18–28. 10.1089/hgtb.2011.034 22428977

[bib72] Bączyk M, Alami NO, Delestrée N, Martinot C, Tang L, Commisso B, Bayer D, Doisne N, Frankel W, Manuel M, (2020) Synaptic restoration by cAMP/PKA drives activity-dependent neuroprotection to motoneurons in ALS. J Exp Med 217: e20191734 10.1084/jem.20191734 32484501 PMC7398175

[bib5] Bang J, Kim HY, Lee H (2016) Optogenetic and chemogenetic approaches for studying astrocytes and gliotransmitters. Exp Neurobiol 25: 205–221. 10.5607/en.2016.25.5.205 27790055 PMC5081467

[bib6] Bataveljic D, Milosevic M, Radenovic L, Andjus P (2014) Novel molecular biomarkers at the blood-brain barrier in ALS. Biomed Res Int 2014: 907545 10.1155/2014/907545 24949481 PMC4037612

[bib7] Benz F, Wichitnaowarat V, Lehmann M, Germano RF, Mihova D, Macas J, Adams RH, Taketo MM, Plate K-H, Guérit S (2019) Low wnt/β-catenin signaling determines leaky vessels in the subfornical organ and affects water homeostasis in mice. Elife 8: e43818 10.7554/elife.43818 30932814 PMC6481993

[bib78] Boillée S, Yamanaka K, Lobsiger CS, Copeland NG, Jenkins NA, Kassiotis G, Kollias G, Cleveland DW (2006) Onset and progression in inherited ALS determined by motor neurons and microglia. Science 312: 1389–1392. 10.1126/science.1123511 16741123

[bib8] Bosco DA, Morfini G, Karabacak NM, Song Y, Gros-Louis F, Pasinelli P, Goolsby H, Fontaine BA, Lemay N, McKenna-Yasek D (2010) Wild-type and mutant SOD1 share an aberrant conformation and a common pathogenic pathway in ALS. Nat Neurosci 13: 1396 10.1038/nn.2660 20953194 PMC2967729

[bib9] Brenner D, Sieverding K, Bruno C, Lüningschrör P, Buck E, Mungwa S, Fischer L, Brockmann SJ, Ulmer J, Bliederhäuser C (2019) Heterozygous Tbk1 loss has opposing effects in early and late stages of ALS in mice. J Exp Med 216: 267–278. 10.1084/jem.20180729 30635357 PMC6363427

[bib74] Caligiuri G (2019) Mechanotransduction, immunoregulation, and metabolic functions of CD31 in cardiovascular pathophysiology. Cardiovasc Res 115: 1425–1434. 10.1093/cvr/cvz132 31119265

[bib10] Chang J, Mancuso MR, Maier C, Liang X, Yuki K, Yang L, Kwong JW, Wang J, Rao V, Vallon M (2017) Gpr124 is essential for blood–brain barrier integrity in central nervous system disease. Nat Med 23: 450 10.1038/nm.4309 28288111 PMC5559385

[bib11] Cho C, Smallwood PM, Nathans J (2017) Reck and Gpr124 are essential receptor cofactors for Wnt7a/Wnt7b-specific signaling in mammalian CNS angiogenesis and blood-brain barrier regulation. Neuron 95: 1056–1073.e5. 10.1016/j.neuron.2017.07.031 28803732 PMC5586543

[bib12] Commisso B, Ding L, Varadi K, Gorges M, Bayer D, Boeckers TM, Ludolph AC, Kassubek J, Müller OJ, Roselli F (2018) Stage-dependent remodeling of projections to motor cortex in ALS mouse model revealed by a new variant retrograde-AAV9. Elife 7: e36892 10.7554/elife.36892 30136928 PMC6125125

[bib13] Corada M, Orsenigo F, Bhat GP, Conze LL, Breviario F, Cunha SI, Claesson-Welsh L, Beznoussenko GV, Mironov AA, Dejana E (2019) Fine-tuning of Sox17 and canonical Wnt coordinates the permeability properties of the blood-brain barrier. Circ Res 124: 511–525. 10.1161/circresaha.118.313316 30591003 PMC6407809

[bib14] Daneman R, Agalliu D, Zhou L, Kuhnert F, Kuo CJ, Barres BA (2009) Wnt/β-catenin signaling is required for CNS, but not non-CNS, angiogenesis. Proc Natl Acad Sci U S A 106: 641–646. 10.1073/pnas.0805165106 19129494 PMC2626756

[bib15] Durkee CA, Araque A (2019) Diversity and specificity of astrocyte-neuron communication. Neuroscience 396: 73–78. 10.1016/j.neuroscience.2018.11.010 30458223 PMC6494094

[bib16] Durkee CA, Covelo A, Lines J, Kofuji P, Aguilar J, Araque A (2019) Gi/o protein‐coupled receptors inhibit neurons but activate astrocytes and stimulate gliotransmission. Glia 67: 1076–1093. 10.1002/glia.23589 30801845 PMC6462242

[bib17] Epperly MW, Fisher R, Rigatti L, Watkins S, Zhang X, Hou W, Shields D, Franicola D, Bayir H, Wang H (2019) Amelioration of amyotrophic lateral sclerosis in SOD1G93A mice by M2 microglia from transplanted marrow. In Vivo 33: 675–688. 10.21873/invivo.11526 31028184 PMC6559904

[bib18] Feng D, Nagy JA, Pyne K, Dvorak HF, Dvorak AM (2004) Ultrastructural localization of platelet endothelial cell adhesion molecule (PECAM-1, CD31) in vascular endothelium. *J Histochem Cytochem* 52: 87–101. 10.1177/002215540405200109 PMID: 14688220. 14688220

[bib19] Forsberg KME, Zhang Y, Reiners J, Ander M, Niedermayer A, Fang L, Neugebauer H, Kassubek J, Katona I, Weis J, (2018) Endothelial damage, vascular bagging and remodeling of the microvascular bed in human microangiopathy with deep white matter lesions. Acta Neuropathol Commun 6: 128 10.1186/s40478-018-0632-z 30470258 PMC6260986

[bib20] Frakes AE, Braun L, Ferraiuolo L, Guttridge DC, Kaspar BK (2017) Additive amelioration of ALS by co‐targeting independent pathogenic mechanisms. Ann Clin Transl Neurol 4: 76–86. 10.1002/acn3.375 28168207 PMC5288461

[bib21] Garbuzova-Davis S, Haller E, Saporta S, Kolomey I, Nicosia SV, Sanberg PR (2007a) Ultrastructure of blood–brain barrier and blood–spinal cord barrier in SOD1 mice modeling ALS. Brain Res 1157: 126–137. 10.1016/j.brainres.2007.04.044 17512910

[bib22] Garbuzova-Davis S, Hernandez-Ontiveros DG, Rodrigues MC, Haller E, Frisina-Deyo A, Mirtyl S, Sallot S, Saporta S, Borlongan CV, Sanberg PR (2012) Impaired blood–brain/spinal cord barrier in ALS patients. Brain Res 1469: 114–128. 10.1016/j.brainres.2012.05.056 22750125

[bib23] Garbuzova-Davis S, Saporta S, Haller E, Kolomey I, Bennett SP, Potter H, Sanberg PR (2007b) Evidence of compromised blood-spinal cord barrier in early and late symptomatic SOD1 mice modeling ALS. PloS One 2: e1205 10.1371/journal.pone.0001205 18030339 PMC2075163

[bib76] Halleskog C, Dijksterhuis JP, Kilander MBC, Becerril-Ortega J, Villaescusa JC, Lindgren E, Arenas E, Schulte G (2012) Heterotrimeric G protein-dependent WNT-5A signaling to ERK1/2 mediates distinct aspects of microglia proinflammatory transformation. J Neuroinflammation 9: 111 10.1186/1742-2094-9-111 22647544 PMC3458933

[bib75] Halleskog C, Mulder J, Dahlström J, Mackie K, Hortobágyi T, Tanila H, Kumar Puli L, Färber K, Harkany T, Schulte G (2011) WNT signaling in activated microglia is proinflammatory. Glia 59: 119–131. 10.1002/glia.21081 20967887 PMC3064522

[bib77] Halleskog C, Schulte G (2013) WNT-3A and WNT-5A counteract lipopolysaccharide-induced pro-inflammatory changes in mouse primary microglia. J Neurochem 125: 803–808. 10.1111/jnc.12250 23534675

[bib24] Hardiman O, Al-Chalabi A, Chio A, Corr EM, Logroscino G, Robberecht W, Shaw PJ, Simmons Z, Van Den Berg LH (2017) Amyotrophic lateral sclerosis. Nat Rev Dis Primers 3: 17071 10.1038/nrdp.2017.85 28980624

[bib25] Hussaini SM, Jun H, Cho CH, Kim HJ, Kim WR, Jang MH (2013) Heat-induced antigen retrieval: An effective method to detect and identify progenitor cell types during adult hippocampal neurogenesis. J Vis Exp 50769 10.3791/50769 PMC3857113 24022759

[bib26] Igarashi Y, Utsumi H, Chiba H, Yamada-Sasamori Y, Tobioka H, Kamimura Y, Furuuchi K, Kokai Y, Nakagawa T, Mori M, (1999) Glial cell line-derived neurotrophic factor induces barrier function of endothelial cells forming the blood-brain barrier. Biochem Biophys Res Commun 261: 108–112. 10.1006/bbrc.1999.0992 10405331

[bib27] Jones ME, Paniccia JE, Lebonville CL, Reissner KJ, Lysle DT (2018) Chemogenetic manipulation of dorsal hippocampal astrocytes protects against the development of stress-enhanced fear learning. Neuroscience 388: 45–56. 10.1016/j.neuroscience.2018.07.015 30030056

[bib28] Jungmann A, Leuchs B, Rommelaere J, Katus HA, Müller OJ (2017) Protocol for efficient generation and characterization of adeno-associated viral vectors. Hum Gene Ther Methods 28: 235–246. 10.1089/hgtb.2017.192 29048971

[bib29] Kisler K, Nelson AR, Rege SV, Ramanathan A, Wang Y, Ahuja A, Lazic D, Tsai PS, Zhao Z, Zhou Y (2017) Pericyte degeneration leads to neurovascular uncoupling and limits oxygen supply to brain. Nat Neurosci 20: 406 10.1038/nn.4489 28135240 PMC5323291

[bib30] Kofuji P, Araque A (2020) G-protein-coupled receptors in astrocyte-neuron communication [published online ahead of print, 2020 Mar 26]. Neuroscience S0306-4522–9. 10.1016/j.neuroscience.2020.03.025 PMC8817509 32224231

[bib31] Kubotera H, Ikeshima-Kataoka H, Hatashita Y, Mascaro ALA, Pavone FS, Inoue T (2019) Astrocytic endfeet re-cover blood vessels after removal by laser ablation. Sci Rep 9: 1263 10.1038/s41598-018-37419-4 30718555 PMC6362239

[bib32] Laksitorini MD, Yathindranath V, Xiong W, Hombach-Klonisch S, Miller DW (2019) Modulation of Wnt/β-catenin signaling promotes blood-brain barrier phenotype in cultured brain endothelial cells. *Sci Rep* 9: 19718 10.1038/s41598-019-56075-w PMID: 31873116 PMCID: PMC6928218. 31873116 PMC6928218

[bib33] LeBlanc NJ, Menet R, Picard K, Parent G, Tremblay M-È, ElAli A (2019) Canonical Wnt pathway maintains blood-brain barrier integrity upon ischemic stroke and its activation ameliorates tissue plasminogen activator therapy. Mol Neurobiol 56: 6521–6521. 10.1007/s12035-019-1539-9 30852795

[bib34] Lee SW, Kim WJ, Choi YK, Song HS, Son MJ, Gelman IH, Kim YJ, Kim KW (2003) SSeCKS regulates angiogenesis and tight junction formation in blood-brain barrier. Nat Med 9: 900–906. 10.1038/nm889 12808449

[bib35] Liebner S, Corada M, Bangsow T, Babbage J, Taddei A, Czupalla CJ, Reis M, Felici A, Wolburg H, Fruttiger M (2008) Wnt/β-catenin signaling controls development of the blood–brain barrier. J Cell Biol 183: 409–417. 10.1083/jcb.200806024 18955553 PMC2575783

[bib36] Liebner S, Dijkhuizen RM, Reiss Y, Plate KH, Agalliu D, Constantin G (2018) Functional morphology of the blood-brain barrier in health and disease. Acta Neuropathol 135: 311–336. 10.1007/s00401-018-1815-1 29411111 PMC6781630

[bib37] Magnus CJ, Lee PH, Atasoy D, Su HH, Looger LL, Sternson SM (2011) Chemical and genetic engineering of selective ion channel–ligand interactions. Science 333: 1292–1296. 10.1126/science.1206606 21885782 PMC3210548

[bib38] Martinez-Silva ML, Imhoff-Manuel RD, Sharma A, Heckman CJ, Shneider NA, Roselli F, Zytnicki D, Manuel M (2018) Hypoexcitability precedes denervation in the large fast-contracting motor units in two unrelated mouse models of ALS. Elife 7: e30955 10.7554/elife.30955 29580378 PMC5922970

[bib39] Matsushita T, Elliger S, Elliger C, Podsakoff G, Villarreal L, Kurtzman G, Iwaki Y, Colosi P (1998) Adeno-associated virus vectors can be efficiently produced without helper virus. Gene Ther 5: 938 10.1038/sj.gt.3300680 9813665

[bib40] Meister S, Storck SE, Hameister E, Behl C, Weggen S, Clement AM, Pietrzik CU (2015) Expression of the ALS-causing variant hSOD1G93A leads to an impaired integrity and altered regulation of claudin-5 expression in an in vitro blood–spinal cord barrier model. J Cereb Blood Flow Metab 35: 1112–1121. 10.1038/jcbfm.2015.57 25853911 PMC4640277

[bib41] Nagai J, Rajbhandari AK, Gangwani MR, Hachisuka A, Coppola G, Masmanidis SC, Fanselow MS, Khakh BS (2019) Hyperactivity with disrupted attention by activation of an astrocyte synaptogenic cue. Cell 177: 1280–1292.e20. 10.1016/j.cell.2019.03.019 31031006 PMC6526045

[bib42] Nielsen S, Nagelhus EA, Amiry-Moghaddam M, Bourque C, Agre P, Ottersen OP (1997) Specialized membrane domains for water transport in glial cells: High-resolution immunogold cytochemistry of aquaporin-4 in rat brain. J Neurosci 17: 171–180. 10.1523/JNEUROSCI.17-01-00171.1997 8987746 PMC6793699

[bib43] Olde Heuvel F, Holl S, Chandrasekar A, Li Z, Wang Y, Rehman R, Förstner P, Sinske D, Palmer A, Wiesner D (2019) STAT6 mediates the effect of ethanol on neuroinflammatory response in TBI. Brain Behav Immun 81: 228–246. 10.1016/j.bbi.2019.06.019 31207335

[bib44] Orr AG, Hsiao EC, Wang MM, Ho K, Kim DH, Wang X, Guo W, Kang J, Yu G-Q, Adame A (2015) Astrocytic adenosine receptor A 2A and G s-coupled signaling regulate memory. Nat Neurosci 18: 423 10.1038/nn.3930 25622143 PMC4340760

[bib45] Ouali Alami NO, Schurr C, Heuvel FO, Tang L, Li Q, Tasdogan A, Kimbara A, Nettekoven M, Ottaviani G, Raposo C (2018) NF‐κB activation in astrocytes drives a stage‐specific beneficial neuroimmunological response in ALS. EMBO J 37: e98697 10.15252/embj.201798697 29875132 PMC6092622

[bib46] Pinzón-Daza ML, Salaroglio IC, Kopecka J, Garzòn R, Couraud PO, Ghigo D, Riganti C (2014) The cross-talk between canonical and non-canonical Wnt-dependent pathways regulates P-glycoprotein expression in human blood-brain barrier cells. *J Cereb Blood Flow Metab* 34: 1258–1269. 10.1038/jcbfm.2014.100 Epub 2014 Jun 4. PMID: 24896565. PMCID: PMC4126086. 24896565 PMC4126086

[bib47] Proffitt KD, Madan B, Ke Z, Pendharkar V, Ding L, Lee MA, Hannoush RN, Virshup DM (2013) Pharmacological inhibition of the Wnt acyltransferase PORCN prevents growth of WNT-driven mammary cancer. Cancer Res 73: 502–507. 10.1158/0008-5472.can-12-2258 23188502

[bib48] Puentes F, Malaspina A, Van Noort JM, Amor S (2016) Non‐neuronal cells in ALS: Role of glial, immune cells and blood‐CNS barriers. Brain Pathol 26: 248–257. 10.1111/bpa.12352 26780491 PMC8029111

[bib49] Pun S, Santos AF, Saxena S, Xu L, Caroni P (2006) Selective vulnerability and pruning of phasic motoneuron axons in motoneuron disease alleviated by CNTF. Nat Neurosci 9: 408 10.1038/nn1653 16474388

[bib50] Roth BL (2016) DREADDs for neuroscientists. Neuron 89: 683–694. 10.1016/j.neuron.2016.01.040 26889809 PMC4759656

[bib51] Rudnick ND, Griffey CJ, Guarnieri P, Gerbino V, Wang X, Piersaint JA, Tapia JC, Rich MM, Maniatis T (2017) Distinct roles for motor neuron autophagy early and late in the SOD1G93A mouse model of ALS. Proc Natl Acad Sci U S A 114: E8294–E8303. 10.1073/pnas.1704294114 28904095 PMC5625902

[bib52] Roudnicky F, Kim BK, Lan Y, Schmucki R, Küppers V, Christensen K, Graf M, Patsch C, Burcin M, Meyer CA, (2020) Identification of a combination of transcription factors that synergistically increases endothelial cell barrier resistance. *Sci Rep* 10: 3886 10.1038/s41598-020-60688-x PMID: 32127614. PMCID: PMC7054428. 32127614 PMC7054428

[bib53] Saxena S, Roselli F, Singh K, Leptien K, Julien J-P, Gros-Louis F, Caroni P (2013) Neuroprotection through excitability and mTOR required in ALS motoneurons to delay disease and extend survival. Neuron 80: 80–96. 10.1016/j.neuron.2013.07.027 24094105

[bib54] Scekic-Zahirovic J, El Oussini H, Mersmann S, Drenner K, Wagner M, Sun Y, Allmeroth K, Dieterlé S, Sinniger J, Dirrig-Grosch S (2017) Motor neuron intrinsic and extrinsic mechanisms contribute to the pathogenesis of FUS-associated amyotrophic lateral sclerosis. Acta Neuropathol 133: 887–906. 10.1007/s00401-017-1687-9 28243725 PMC5427169

[bib55] Scekic-Zahirovic J, Sendscheid O, El Oussini H, Jambeau M, Sun Y, Mersmann S, Wagner M, Dieterle S, Sinniger J, Dirrig-Grosch S, (2016) Toxic gain of function from mutant FUS protein is crucial to trigger cell autonomous motor neuron loss. EMBO J 35: 1077–1097. 10.15252/embj.201592559 26951610 PMC4868956

[bib56] Stamatovic SM, Keep RF, Kunkel SL, Andjelkovic AV (2003) Potential role of MCP-1 in endothelial cell tight junction “opening”: Signaling via Rho and Rho kinase. J Cell Sci 116: 4615–4628. 10.1242/jcs.00755 14576355

[bib79] Simard M, Nedergaard M (2004) The neurobiology of glia in the context of water and ion homeostasis. Neuroscience 129: 877–896. 10.1016/j.neuroscience.2004.09.053 15561405

[bib57] Stamatovic SM, Dimitrijevic OB, Keep RF, Andjelkovic AV (2006) Protein kinase Calpha-RhoA cross-talk in CCL2-induced alterations in brain endothelial permeability. J Biol Chem 281: 8379–8388. 10.1074/jbc.M513122200 16439355

[bib58] Stamatovic SM, Keep RF, Wang MM, Jankovic I, Andjelkovic AV (2009) Caveolae-mediated internalization of occludin and claudin-5 during CCL2-induced tight junction remodeling in brain endothelial cells. J Biol Chem 284: 19053–19066. 10.1074/jbc.M109.000521 19423710 PMC2707189

[bib59] Stenman JM, Rajagopal J, Carroll TJ, Ishibashi M, McMahon J, McMahon AP (2008) Canonical Wnt signaling regulates organ-specific assembly and differentiation of CNS vasculature. Science 322: 1247–1250. 10.1126/science.1164594 19023080

[bib60] Torres VI, Godoy JA, Inestrosa NC (2019) Modulating Wnt signaling at the root: Porcupine and Wnt acylation. Pharmacol Ther 198: 34–45. 10.1016/j.pharmthera.2019.02.009 30790642

[bib61] Tran KA, Zhang X, Predescu D, Huang X, Machado RF, Göthert JR, Malik AB, Valyi-Nagy T, Zhao Y-Y (2016) Endothelial β-catenin signaling is required for maintaining adult blood–brain barrier integrity and central nervous system homeostasis. Circulation 133: 177–186. 10.1161/circulationaha.115.015982 26538583 PMC4814374

[bib63] Vogelmann R, Nelson WJ (2005) Fractionation of the epithelial apical junctional complex: Reassessment of protein distributions in different substructures. Mol Biol Cell 16: 701–716. 10.1091/mbc.e04-09-0827 15548593 PMC545905

[bib64] Wang Y, Cho C, Williams J, Smallwood PM, Zhang C, Junge HJ, Nathans J (2018) Interplay of the Norrin and Wnt7a/Wnt7b signaling systems in blood–brain barrier and blood–retina barrier development and maintenance. Proc Natl Acad Sci U S A 115: E11827–E11836. 10.1073/pnas.1813217115 30478038 PMC6294914

[bib65] Watanabe-Matsumoto S, Moriwaki Y, Okuda T, Ohara S, Yamanaka K, Abe Y, Yasui M, Misawa H (2018) Dissociation of blood-brain barrier disruption and disease manifestation in an aquaporin-4-deficient mouse model of amyotrophic lateral sclerosis. Neurosci Res 133: 48–57. 10.1016/j.neures.2017.11.001 29154923

[bib66] Wiesner D, Tar L, Linkus B, Chandrasekar A, Olde Heuvel F, Dupuis L, Tsao W, Wong PC, Ludolph A, Roselli F (2018) Reversible induction of TDP-43 granules in cortical neurons after traumatic injury. Exp Neurol 299: 15–25. 10.1016/j.expneurol.2017.09.011 28941811

[bib67] Winkler EA, Sengillo JD, Sagare AP, Zhao Z, Ma Q, Zuniga E, Wang Y, Zhong Z, Sullivan JS, Griffin JH (2014) Blood–spinal cord barrier disruption contributes to early motor-neuron degeneration in ALS-model mice. Proc Natl Acad Sci U S A 111: E1035–E1042. 10.1073/pnas.1401595111 24591593 PMC3964055

[bib68] Winkler EA, Sengillo JD, Sullivan JS, Henkel JS, Appel SH, Zlokovic BV (2013) Blood–spinal cord barrier breakdown and pericyte reductions in amyotrophic lateral sclerosis. Acta Neuropathol 125: 111–120. 10.1007/s00401-012-1039-8 22941226 PMC3535352

[bib69] Wu GY, Deisseroth K, Tsien RW (2001) Activity-dependent CREB phosphorylation: Convergence of a fast, sensitive calmodulin kinase pathway and a slow, less sensitive mitogen-activated protein kinase pathway. Proc Natl Acad Sci U S A 98: 2808–2813. 10.1073/pnas.051634198 11226322 PMC30221

[bib70] Zhong Z, Deane R, Ali Z, Parisi M, Shapovalov Y, O’Banion MK, Stojanovic K, Sagare A, Boillee S, Cleveland DW (2008) ALS-causing SOD1 mutants generate vascular changes prior to motor neuron degeneration. Nat Neurosci 11: 420 10.1038/nn2073 18344992 PMC2895310

[bib71] Zhong Z, Ilieva H, Hallagan L, Bell R, Singh I, Paquette N, Thiyagarajan M, Deane R, Fernandez JA, Lane S (2009) Activated protein C therapy slows ALS-like disease in mice by transcriptionally inhibiting SOD1 in motor neurons and microglia cells. J Clin Invest 119: 3437–3449. 10.1172/JCI38476 19841542 PMC2769191

