# Supplementary material for: Multiplexed chemogenetics in astrocytes and motoneurons restore blood–spinal cord barrier in ALS
Source: Life Sci Alliance. 2020 Sep 8;3(11):e201900571. doi: 10.26508/lsa.201900571 (PMC7479971; doi:10.26508/lsa.201900571)
Supplement: Supplementary file 1 [file LSA-2019-00571_Supplemental_Data_1.docx]

**Supplementary Material**

*Supplementary methods*

*AAV production:* Production of AAVs was carried out as previously reported (Commisso et al, 2018) using a three plasmid system (Matsushita et al, 1998) in adherent cell culture. For this purpose, HEK 293t cells were transfected with polyethylenimine and harvested after three days of incubation. Transfection and subsequent purification of viral particles from cells and supernatant was performed according to the protocol by Jungmann et al, 2017. Quantification was accomplished by QPCR using primers targeting the inverted terminal repeats (Aurnhammer et al, 2011). All AAV suspension was titrated at 5-9*1013 genomes/ml.

*Intraspinal injection of AAV:* For single-virus injections, 1 µl of viral suspension was diluted with 0.5ul of 1% Fast-Green solution (in PBS+ Ca2+/Mg2+, Invitrogen). When multiple AAVs were injected (for multiplexed chemogenetics), a mix of 0.5 µl of each virus was diluted with 0.5µl of 1% Fast-Green. Intraspinal injection of AAV was performed as previously reported (Saxena et al, 2013). Briefly, animals were administered Buprenorphine (0.05 mg/kg; Reckitt Benckiser healthcare Ltd, Berkshire, UK) and Meloxicam (1.0 mg/kg; Metacam, Boehringer-Ingelheim, Ridgeland, CT) 30 min before isoflurane anesthesia (4% in O2 at 800 ml/min). Animals were positioned in a stereotaxic frame (Bilaney Consultants GmbH D-40211 Düsseldorf, Germany), and the dorsal skin was shaved and incised at the low-thoracic/lumbar level. Dorsal fascia was resected and paraspinal muscles were blunt-dissected to expose the underlying vertebral laminae. The interspinous ligaments were cut between T11 and T13, and the vertebral dorsal laminae were sectioned medially at the level of the spinous process and laterally at the level of the processus mammillaris. The vertebral laminar flap was then lifted to access the dura without damaging the underlying spinal cord; dura meninges were opened using a G33 needle. Using the central dorsal artery as reference, injections were performed at one/two sites (spaced 0.5-1mm) in the right or left hemicord at the coordinates y=+0.30; z=-0.45. A total of 1μl of viral suspension was injected during 15 min with a glass capillary coupled to a Picospritzer-III apparatus in 8-10 msec pulses. Paraspinal muscles were repositioned and scalp skin was then stitched with Prolene 7.0 surgical threads. Animals were transferred for recovery in single cages with facilitated access to water and food, and monitored for eventual neurological impairment for the following 72h. During the recovery period, animals were administered buprenorphine twice per day and Meloxicam once per day.

*Histology and immunostaining*: For the preparation of spinal cord samples for histological evaluation, mice were deeply anesthetized with 1 mg/kg body weight ketamine chlorhydrate and 0.5 mg/kg body weight xylazine and transcardially perfused with 2.0 ml/g ice-cold 0.1 M PBS followed by 2.5-3 ml/g of 4% ice-cold PFA (pH 7.4). After perfusion, spinal cord samples were quickly dissected and post-fixed in 4% PFA for 18h at 4°C, washed in PBS, and cryoprotected in 30% Sucrose for 24-36h. Samples were then embedded in OCT (TissueTek) and 40µm-thick sections were obtained by a Leica CM1950 cryostat, set at -18°C. The sections were collected serially and washed in 0.1 M PBS. Free-floating spinal cord sections were then incubated in a 10 mM sodium citrate buffer (pH 8.0) for antigen retrieval (Hussaini et al, 2013) at 80°C for 2h in a water bath. After the unmasking procedure, the free-floating sections were blocked with 3% BSA and 0.3%Triton for 2h at RT and immunostained with the appropriate antibodies combination (see Supplementary table 1- diluted in the blocking buffer) for 48-72h at 4°C, followed by washing steps in PBS (30 min x 3) and incubation with the appropriate combination of secondary antibodies (see Supplementary table 2)  for 2h at RT. After washing, spinal cord sections were mounted with ProLong Gold Antifade (ThermoFisher).

*Single-molecule in situ mRNA hybridization*: Detection of mRNA in situ was performed as previously reported (Olde-Heuvel et al, 2019) and according to the manufacturer's recommendation (Acd Bio). Briefly, the sections were mounted on microscopic slides and air dried at -20°C. Autofluorescence quenching was performed by treating the samples with 0.1M glycine in PBS for 15 min, followed by a 3 min antigen retrieval, washed twice in dH20 and once in ethanol. A pretreatment step with reagent III was performed for 20 min, followed by washing in dH20. The probes (either against Wnt5a or against Wnt7a) were hybridised for 4h and 30 min at 40°C followed by two washing steps of 2 min each with a washing buffer. 3 Amplification steps were performed each followed by two washes. A last detection step with a fluorescent signal was performed for 45 min followed by two wash steps of 10 min each. The sections were then blocked in a blocking buffer (10% BSA, 0,3% Triton in 1x PBS) for 1h at RT and incubated with primary antibody anti-GFP (1:500), anti-GFAP (1:300), and anti-collagen-IV (1:200 overnight. The sections were washed 3 times for 30 min in PBS and incubated in secondary antibody donkey anti-chicken, donkey anti-mouse and donkey anti-rabbit respectively (1:500) for 2h, followed by 3 washes for 30 min each in PBS. The sections were finally mounted with fluorogold prolong antifade mounting medium (invitrogen) and acquired with a confocal microscope LSM-710 (Carl Zeiss AG).

*Confocal imaging and image analysis:* Confocal images were acquired using a LSM-710 (Carl Zeiss AG) inverted microscope fitted with a 20X air or 40X or 63X oil objective. Images were captured in a 12-bit format at a resolution of 1024x1024 pixels (x-y) and a theoretically optimal optical section thickness (z). Imaging parameters were set in order to obtain a minimal signal intensity for the immunostained antigen >150 (range 0-4095 in 12-bits images) while avoiding saturation. Imaging parameters were kept constant across imaging sessions and samples. All images were acquired in correspondence with the gray matter of the ventral horn of the spinal cord (Fig 1F).

For the quantification of disease burden (misfolded SOD1, LC3A), the images were imported using FIJI image analysis software. Confocal stacks (5-9 1µm-thick optical sections, 20x objective) were collapsed in a maximum-intensity projection, regions of interest (ROIs) were manually traced around MN (using the VAChT staining as reference), and the integrated mean gray value was obtained. Fluorescence intensity was expressed in arbitrary units (a.u) corresponding to the grayscale value (in 12-bits images, ranging from 0 to 4095). For the quantification of p62 burden, first the cytoplasm area of individual MN was determined by manually tracing the cellular contour (using VAChT as reference and excluding the nucleus), then the images were thresholded (to segment the bright p62 aggregates), and the cumulative area of p62 aggregates was computed; p62 burden was then quantified as the ratio between the area occupied by the p62 aggregates and the area of the cytoplasm.

For the quantification of the structural disruption of the BSCB, we considered a region of interest in the ventral spinal cord. We traced the total length of vessels in the ROI, identified by collagen-IV^+^ staining, and we then traced the length of vascular segments in which the claudin-5 ribbon appeared disrupted or fragmented (“gaps”). We computed the ratio between the cumulative gaps length along claudin-5 ribbons and the total length of the vessels, as previously reported (Ouali Alami et al, 2018).

For the quantification of the astrocytic end-feet coverage of spinal cord vessels, blood vessels were first identified using collagen-IV staining and a region of interest corresponding to the vessels was manually traced. The surface of the vessel occupied by GFP-positive processes was then quantified. For quantitative analysis, a minimum of 8-10 artifact-free sections per mouse were analyzed.
